# Designing a Visual Analytics Tool to Support Data Analysis Tasks of Digital Mental Health Interventions: Case Study

**DOI:** 10.2196/64967

**Published:** 2025-07-02

**Authors:** Gyuwon Jung, Heejeong Lim, Kyungsik Han, Hyungsook Kim, Uichin Lee

**Affiliations:** 1 School of Computing KAIST Daejeon Republic of Korea; 2 Graduate School of Data Science KAIST Daejeon Republic of Korea; 3 Department of Data Science Hanyang University Seoul Republic of Korea; 4 Department of Artificial Intelligence Hanyang University Seoul Republic of Korea; 5 Hanyang Digital Healthcare Center Hanyang University Seoul Republic of Korea

**Keywords:** digital health interventions, visual analytics, data analysis tasks, user characteristics, user engagement, effectiveness, mental health, observational data, user experience, human-data interaction

## Abstract

**Background:**

Digital health interventions (DHIs) are widely used to manage users’ health in everyday life through digital devices. The use of DHIs generates various data, such as records of intervention use and the status of target symptoms, providing researchers with data-driven insights for improving these interventions even after deployment. Although DHI researchers have investigated these data, existing analysis practices have been fragmented, limiting a comprehensive understanding of the data.

**Objective:**

We proposed an analysis task model to help DHI researchers analyze observational data from a holistic perspective. This model was then used to prototype an interactive visual analytics tool. We aimed to evaluate the suitability of the model for DHI data analysis and explore task support using a visual analytics tool.

**Methods:**

We constructed a data analysis task model using 3 key components (ie, user grouping criteria) for DHI data analysis: user characteristics, user engagement with DHIs, and the effectiveness of DHIs on target symptoms based on comparisons before and after the intervention. On the basis of this model, we designed Maum Health Analytics, a medium-fidelity prototype of an interactive visual analytics tool. Each feature of the prototype was mapped one-to-one to the analysis task described in the model. To investigate whether the proposed model adequately reflects real-world DHI analysis needs, we conducted a preliminary user study with 5 groups of researchers (N=15). Participants explored the tool through scenario-based analysis tasks using in-the-wild data collected from a mobile DHI service targeting depressive symptoms. Following the session, we conducted interviews to assess the appropriateness of the defined tasks and the usability and practical utility of the visual analytics tool.

**Results:**

DHI researchers responded positively to both the analysis task model and the visual analytics tool. In the interviews, participants noted that the tool supported the identification of users who needed additional care, informed content recommendations, and helped analyze intervention effectiveness in relation to user characteristics and engagement levels. They also appreciated the tool’s role in simplifying analytic tasks and supporting communication across multidisciplinary teams. Additional suggestions included improvements for continuity across tasks and more detailed engagement metrics.

**Conclusions:**

We proposed an analysis task model and designed an interactive visual analytics tool to support DHI researchers. Our user study showed that the model allows a holistic investigation of DHI data by integrating key analysis components and that the prototype tool simplifies analytic tasks and enhances communication among researchers. As DHIs grow, the proposed model and tool can effectively meet the data analysis requirements of researchers and improve efficiency.

## Introduction

### Background

Digital devices, such as smartphones and wearables, are extensively used in health care to deliver interventions beyond traditional medical settings [1]. These interventions, known as digital health interventions (DHIs), are designed to address various health issues and promote healthy behaviors, such as physical activity and smoking cessation, or to manage chronic conditions, such as depression and diabetes, in daily life [2,3].

DHIs use various intervention strategies, including behavior change techniques, which have proven to be effective in altering health behaviors [4-6]. These techniques include monitoring user behavior, setting goals, providing social support, and incorporating gamification. Furthermore, DHIs can be classified into different categories, such as digital health, medicine, and therapeutics, based on clinical evidence and real-world outcomes [7]. Previous studies [8-11] have investigated the delivery procedures of DHIs, identified barriers in their delivery paths, and determined the opportune moments for providing DHIs. All these studies share the common objective of effectively improving user behavior or symptoms targeted by DHIs.

DHIs are commonly designed as mobile apps, such as those in mobile health, which enable individuals to install and use them on smart devices. When users engage with the features and content offered by DHIs, various data are collected. For instance, several types of log data are passively gathered during user interactions with DHIs. Most of these log data consist of detailed records of user activities, including the frequency of DHI app use; individual DHI content accessed; and user interactions, such as taps, swipes, and text entries, within particular intervention content [12]. In addition, some data are actively provided by users through manual inputs, which are essential for capturing information that may be challenging to track using passive log data alone. These data often include basic user information, such as sociodemographic details, and periodic self-reports on their physical or mental health status.

Previous studies have performed various analyses to better understand the data collected from DHIs and assess either user engagement with the intervention content or the effectiveness of the interventions in supporting target users. Researchers studying DHIs have explored user engagement with interventions, assessing either subjective experiences about how immersed users are or objective behaviors, such as use frequency [11]. When quantitatively assessing engagement, researchers can analyze macroindicators, such as the number of log-ins to the DHI, frequency of content access, time spent, sequence of content use, as well as microindicators, such as the number of clicks and swipes [13-16]. A recent study [17] suggested similar metrics for measuring engagement, categorizing them into individual-level metrics, such as launch counts, use durations, and long-term use patterns, and population-level metrics, including the ratio of users who open the app at least once and the number of completers.

In addition, researchers have evaluated the effectiveness of DHIs on target symptoms by comparing health status before and after the use of DHIs in natural settings. The evaluation of DHIs typically progresses from measuring efficacy with a small group of users in controlled environments (eg, randomized controlled trials) to assessing effectiveness with a large group of users in uncontrolled environments [1]. By evaluating effectiveness without predefined treatment and control groups, researchers can investigate whether DHIs can yield the intended health changes or real-world evidence by analyzing real-world data (RWD), although numerous confounding factors exist [18,19]. Several metrics, such as user engagement and DHI effectiveness, can indicate how actively users have used DHIs and whether they have experienced improved health conditions, thus providing reasonable criteria for evaluating DHIs. A relevant example is the SilverCloud platform proposed by Doherty et al [20], which explores ways to encourage users to engage more actively in online mental health interventions. Using this platform, researchers have proposed approaches for visualizing log data to understand the engagement levels of individuals or groups of users [21] and predict the clinical outcomes of interventions using machine learning techniques [22].

Nevertheless, existing analyses of DHI data have often been performed separately based on individual researchers’ interests, which limits their ability to understand data from various perspectives. Consequently, researchers may miss meaningful relationships among various indicators that can be extracted from DHI data. Moreover, the lack of a comprehensive analysis of DHI use patterns and changes in health status makes it challenging to determine how to improve DHIs according to the needs and preferences of specific user groups. Given that data analyses are repeated throughout the development and evaluation life cycle of DHIs [23], it is necessary to integrate the diverse analytical approaches that researchers can use with DHI data.

### Objectives

Therefore, we proposed an analysis task model to help DHI researchers analyze data from a holistic perspective, enabling them to uncover interactions and patterns that are not visible when these factors are analyzed independently. We constructed this model based on common analysis practices observed in previous DHI research, particularly those identified in a meta-analytic review by Moshe et al [24].

To achieve this goal, we developed an analysis task model to help researchers explore DHI data from multiple perspectives. This model guided us to implement a prototype for an interactive visual analytics tool named Maum Health Analytics, which was designed to support the practical application of the model. The tool was evaluated using RWD collected from Maum Health, a mobile DHI service for individuals experiencing depressive symptoms.

This study aimed to evaluate whether the proposed analysis task model is suitable for DHI data analysis and explore how visual analytics can support such tasks. Our preliminary user study identified several benefits of the Maum Health Analytics tool from the human-computer interaction perspective. We found that the proposed tool helped DHI research teams investigate user engagement and the effectiveness of the Maum Health DHI service from various angles, gain insights for recommending intervention content, and better understand users who may need additional care. Furthermore, Maum Health Analytics facilitated communication among various stakeholders, streamlined repetitive analysis tasks, and exhibited potential for integration with existing analytic systems.

Overall, our contribution can be summarized as follows:

1. We proposed an analysis task model for DHIs, aimed at assisting researchers in understanding DHI data from diverse perspectives.
2. We designed an interactive visual analytics tool based on the proposed model and explored its feasibility and design implications to better support researchers in analyzing DHI data.

## Methods

This section describes the development of the analysis task model, the visual analytics tool, as well as the procedure of the preliminary user study conducted with the prototype. The subsequent sections present the quantitative and qualitative study results and discuss their implications for future research and the design of DHI analytics.

### Analysis Task Model

#### Overview

As part of our previous work on DHIs [25], we conducted an in-depth analysis of RWD collected from a mobile DHI service named Maum Health, which supports individuals with depression. Through this process, we identified recurring analytical needs among DHI researchers, motivating us to design a dedicated visual analytics tool for this study. Within this service, users could assess their depression symptoms using the 10-item Center of Epidemiologic Studies Depression Scale (CES-D-10) questionnaire [26] and access intervention content, such as art therapy (named Mandala), physical activity promotion (named Geunsimtapa), and cognitive-emotional games (named Finding Blue) to alleviate symptoms. Following a 3-month deployment of the DHI, we analyzed depression states and content use logs stored on the server to extract data-driven insights to enhance the intervention service.

As a multidisciplinary DHI research team comprising intervention content designers, clinicians, and system developers, our analysis addressed several research questions, including differences in depression changes based on user characteristics (eg, initial depression level) and the association between DHI use and changes in depression. During the DHI data analysis, we made 3 key observations regarding the analysis tasks.

First, DHI researchers primarily focused on exploring user engagement with DHIs and the effectiveness of these interventions when evaluating existing DHI services. They determined that the DHI service can be considered as well designed if it is used more actively and if it leads to improvements in target symptom levels compared with before its use. In addition, given that the DHI was deployed to assess whether it supports the achievement of the intended outcome (ie, improving the depressive state) in the real world, researchers usually measure the effectiveness of a DHI instead of its efficacy. Unlike efficacy, which is typically measured in clinical trials involving random user assignments to control and treatment groups (controlled research setting), effectiveness is evaluated using data from observational studies, allowing researchers to observe how interventions are performed in natural settings (uncontrolled nonresearch setting) [1]. This measurement is relevant to RWD in health care and medical domains [18], where data are collected without randomly assigning participants to specific treatment conditions, and researchers conduct observational studies [27,28].

Second, researchers have attempted to investigate whether certain groups of users would exhibit higher (or lower) levels of the aforementioned metrics, aiming to examine the relationships between user groups and the metrics. They analyzed the engagement and effectiveness of user groups specified based on certain criteria and compared them across different groups [8]. Although these analyses are valuable, they are often conducted separately, limiting a comprehensive understanding of how different factors, such as user characteristics, engagement levels, and effectiveness, interact. This fragmented approach can result in missing critical insights from a holistic perspective.

To address this gap, we constructed an analysis task model that aimed to integrate these typical analysis task components and facilitate a more thorough understanding of DHI data. Specifically, this model was constructed based on a meta-analytic review by Moshe et al [24], which assessed multiple studies on DHIs developed to address depressive symptoms. The review outlined four factors that previous studies analyzed regarding their association with the effectiveness of DHIs: (1) the characteristics of participants, (2) the presence of guidance in using DHIs, (3) engagement with DHIs, and (4) study design and quality. Because several existing studies have performed similar analyses [24], we determined that these analysis tasks were common and should be integrated into the proposed model. However, we excluded two factors from the proposed model: the presence of guidance in using DHIs and study design and quality. This decision was made because our DHI service did not involve any human expert support, and we did not aim to demonstrate the efficacy of DHIs through clinical trials.

Consequently, we constructed our analysis task model with three key components: (1) user characteristics, (2) user engagement with DHIs, and (3) the effectiveness of DHIs on target symptoms. On the basis of these key components, we identified a set of exploratory analysis tasks by referring to the practices of existing studies, as explained in subsequent sections. These tasks are intended to examine each component and explore how they relate to segmented user groups. For instance, the model supports not only the analysis of overall effectiveness across all users but also comparisons across user subgroups segmented by user characteristics or engagement levels. In this model, effectiveness is treated as an outcome variable to be examined within specific segments instead of as a factor used to define those segments. Although the model enables the exploration of the relationships among components within specific user segments, it does not assume causal or recursive dependencies. Instead, it provides a structured framework for performing an in-depth analysis of specific user segments.

**Figure 1:**
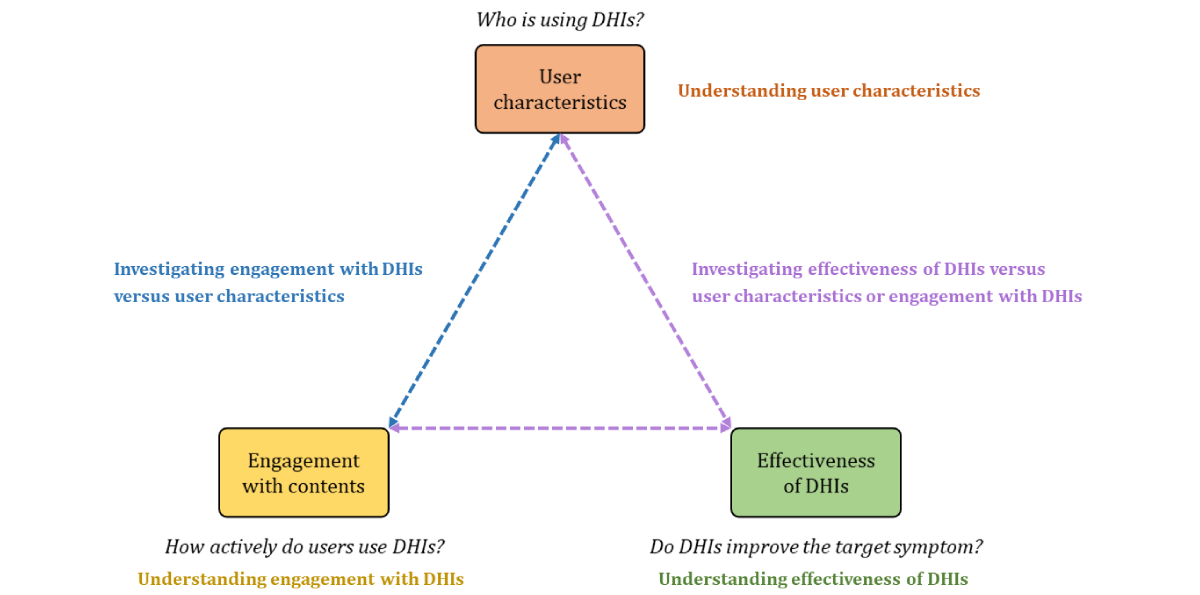
Overview of the proposed analysis task model for digital health intervention (DHI) data.

The overall structure of the proposed model is illustrated in Figure 1, and further details of each component and the associated analysis tasks are described in the subsequent subsections and Table 1.

**Table 1:** Mapping of model-defined analysis tasks to interface, visualizations, and interactions in the Maum Health Analytics tool.

Model components, interface, and task IDs			Task description		Visualization and interactions
**User characteristics (interface: User page)**					
	T1.1	Track the number of users over time		Line chart	
	T1.2	View user distribution by characteristics		Bar chart	
	T1.3	Explore individual user records		Table; filtering (by user characteristics)	
**User engagement with DHIs^a^ (interface: Engagement page)**					
	T2.1	View engagement level across all users		Histogram	
	T2.2	View engagement level across user subgroups		Histogram; filtering (by user characteristics)	
	T2.3	Compare the engagement level among user subgroups		Histogram; group selection (by user characteristics)	
	T2.4	View user characteristics across engagement subgroups		Bar chart; filtering (by user engagement)	
**Effectiveness of DHIs (interface: Effectiveness page)**					
	T3.1	View effectiveness across all users		Histogram	
	T3.2	View effectiveness across user and engagement subgroups		Histogram; filtering (by user characteristics and engagement)	
	T3.3	Compare effectiveness across user and engagement subgroups		Histogram; group selection (by user characteristics and engagement)	
	T3.4	View user or engagement across effectiveness subgroups		Bar chart; filtering (by effectiveness)	

^a^DHI: digital health intervention.

#### User Characteristics

Understanding the characteristics of DHI users is crucial because different user groups may lead to differences in DHI use behaviors and changes in target symptoms. As demonstrated in a large-scale cross-study evaluation by Pratap et al [29], user characteristics, such as gender, age, and geographic or racial or ethnic diversity, may influence the sustained use of DHIs. Similarly, other studies have shown that these characteristics could impact dropout rates from DHIs and changes in outcomes [30,31].

User characteristics for DHI data analysis can vary, including demographics (eg, age, gender, and ethnicity), physical states (eg, height and weight), and psychological states (eg, mood, depression, stress, and motivation) [11]. In addition, factors such as personality traits, digital health literacy, and the availability of time and space for using DHIs can be considered important constructs of user characteristics [9].

Regarding user characteristics, the proposed model comprises 3 analysis tasks, identified based on a review of existing DHI research and the authors’ deployment experiences (as illustrated in the Methods section). First, researchers track the changes in the number of DHI users over time (task T1.1). This task is essential to understanding the overall adoption and sustained use of DHI services. Researchers can assess whether the DHI services are effectively used by monitoring the number of enrolled and active users. Next, researchers examine the distribution of DHI users based on their characteristics (eg, gender, age, and baseline symptom levels), as suggested in previous studies (task T1.2). This task involves sorting and filtering users based on various characteristics to explore the composition of the user population. It helps to identify trends and patterns in user engagement and the demographic reach of the DHI service. Finally, researchers review the detailed records of individual DHI users belonging to specific groups determined by user characteristics (task T1.3). This task allows for a more granular analysis, providing insights into the behavior and engagement levels of users within specific segments of the population. By following these tasks, researchers can gain comprehensive insights into who uses their DHI services through diverse visualizations, ranging from a broad overview to detailed individual records.

#### User Engagement With DHIs

User engagement is an essential metric in DHI research, as highlighted in previous studies. This component is crucial not only for understanding the current activeness of users interacting with DHIs but also for exploring strategies to maintain and enhance their sustained use [32,33]. Low engagement levels in DHIs are analogous to situations in which patients do not properly take medications. Therefore, this metric should be closely monitored to assist DHI users in maintaining their use until desired health outcomes are achieved.

As reviewed by Pham et al [34], previous studies have used various indicators to measure engagement levels, including the frequency of log-ins, accessed DHI features and modules, and duration of DHI use. Among these metrics, we included the frequency (ie, launch counts) and duration (ie, use time) of DHI content use in the proposed analysis task model, as they were commonly used in existing studies [11,35-37]. Furthermore, if a structured activity exists within each intervention, its completion level can be measured and evaluated as a detailed user engagement metric [38].

For user engagement with DHIs, we included 4 analysis tasks in the model. First, researchers assess the engagement level of each DHI content item across all users (task T2.1). This task involves evaluating various engagement metrics for each DHI content item, such as launch counts, use time, and completion levels, as highlighted in previous studies [11]. This provides an overview of how each content item is used.

However, considering that user characteristics may influence engagement with DHIs, researchers assess the engagement level of each DHI content item for user groups specified by user characteristics (task T2.2). This task allows for a segmented analysis based on attributes, such as age, gender, or baseline symptom levels, which helps understand how different user groups interact with DHI content. Furthermore, they compare the engagement level of each DHI content item across different user groups (task T2.3). For instance, to determine whether age affects engagement levels, researchers can compare the distribution of engagement metrics among DHI users of different age groups. This comparative analysis helps identify patterns and variations in engagement across user characteristics.

This group-level analysis can also be performed in reverse; researchers assess the user characteristics distribution for a user group where they attain a certain level of engagement (task T2.4). By performing these analytical tasks, DHI researchers can evaluate the extent to which DHI content is actively used, identify differences in engagement levels among various user groups, and understand the distribution of user characteristics at certain engagement levels.

#### Effectiveness of DHIs

As the primary objective of DHIs is to improve target symptoms, evaluating their effectiveness is necessary. To evaluate the effectiveness of DHIs in real-world settings, researchers analyze the changes in depression symptoms by comparing self-reported symptom levels before and after DHI use. This pre-post comparison allows us to determine whether the intervention has led to a statistically significant improvement in target symptoms, thus estimating the effectiveness of DHIs. As explained in the previous section, effectiveness is measured in an uncontrolled setting to observe how the DHI influences the target symptoms in natural environments. For depression assessment, the following instruments are widely used [39,40]: the 9-item Patient Health Questionnaire [41], the CES-D [42], the Beck Depression Inventory—II [43], and the Patient-Reported Outcomes Measurement Information System [44]. These assess depressive symptoms through items targeting self-reported negative emotions, self-perception, social interaction, and diminished positive affect.

Similar to the analysis of user engagement with DHIs, the analysis task model included 4 tasks for evaluating the effectiveness of the DHIs. The first task is to assess the changes in depression levels across all users based on their self-reported depression states (task T3.1). This task provides an overall measure of the impact of the DHI on target symptoms.

The next task is to assess the changes in depression levels for the user groups (task T3.2) and compare these changes across different user groups (task T3.3). As illustrated previously, user characteristics and engagement levels can influence the effectiveness of DHIs. Hence, the user groups in tasks T3.2 and T3.3 can be specified based on user characteristics or the engagement level of each DHI content item. For instance, effectiveness can be evaluated for a user group comprising women who use a specific DHI content for more than an hour, or it can be compared among user groups divided by age. This segmented and comparative analysis helps to identify how the effectiveness of DHIs varies across user characteristics and engagement levels.

Group-level analysis can be performed in reverse, such as task T2.4; researchers assess user characteristics and engagement level distribution for a user group where they attain a certain change in depression levels (task T3.4). This task is crucial for understanding user characteristics and engagement behaviors associated with significant changes in depression levels. These tasks assist researchers in exploring the effectiveness of DHIs from multiple perspectives. They allow for the analysis of effectiveness while considering moderating factors, thereby enabling a nuanced understanding of how various user characteristics and engagement levels influence outcomes. Moreover, they provide methods to tailor DHIs to different users based on the observed effectiveness across diverse user groups.

### Interactive Visual Analytics

We designed Maum Health Analytics, a prototype of an interactive visual analytics tool, to facilitate researchers in conducting DHI data analysis tasks from multiple perspectives. This prototype was developed based on the analysis task model proposed in the previous section, with each feature directly mapped to a specific analytical task defined in the model.

The primary goal of this prototype was to evaluate whether the proposed model adequately supported the typical analysis workflow used by DHI researchers. To achieve this, we developed a medium-fidelity prototype that implemented the tasks defined in the model. Rather than building a complete system, we focused on creating a functional prototype suitable for the initial evaluation.

The prototype was created using Figma and designed to enable users to explore DHI data through task-specific features embedded in predefined analysis scenarios. The development followed an iterative design process. We created an initial version that mapped model-defined tasks to concrete interface features and functionalities. This prototype was thereafter refined through multiple feedback sessions with 2 domain experts who were clinicians with experience in developing their own DHIs and analyzing real-world DHI data. They participated in expert heuristic evaluations and provided feedback on the clarity of the task flow, adequacy of the visual representations, and interpretability of the analytic results. On the basis of their input, we made several adjustments to the prototype to improve its usability and relevance. In the final version, we incorporated the actual analysis results derived from the RWD collected through our previously deployed DHI, Maum Health. This addition provided users with a realistic context during the preliminary user study and allowed us to better evaluate the usability of the tool.

Each analysis task defined in the model was mapped to a corresponding interface component and visualized in the prototype. Table 1 summarizes the mapping, including the key visual elements and interaction types used to support each task.

Consequently, Maum Health Analytics features 3 main pages. Each page corresponds to 1 of the 3 key analysis components: user characteristics, user engagement with DHIs, and the effectiveness of DHIs. On each page, researchers can select the specific user group conditions that they wish to investigate, and the tool displays the analyzed results interactively. These results are primarily presented through visual elements, such as bar charts, line charts, tables, and histograms, aiding researchers in quickly grasping the overall trends or differences. Moreover, detailed analysis results, such as statistical testing, are provided together to assist researchers in interpreting these findings.

To enhance usability, we included a tag feature throughout the visual analytics tool. Tagging allows the development of researcher-defined groups. For instance, a researcher can create a tag for a user group characterized by high engagement levels and severe initial depression. Once saved, these tags enable quick and easy access to researcher-defined user groups and function as custom favorite lists. This allows researchers to efficiently revisit and analyze the same user groups without having to redefine the conditions, streamlining the data analysis process with Maum Health Analytics.

In the subsequent section, we provide brief explanations for each page of the Maum Health Analytics tool.

**Figure 2:**
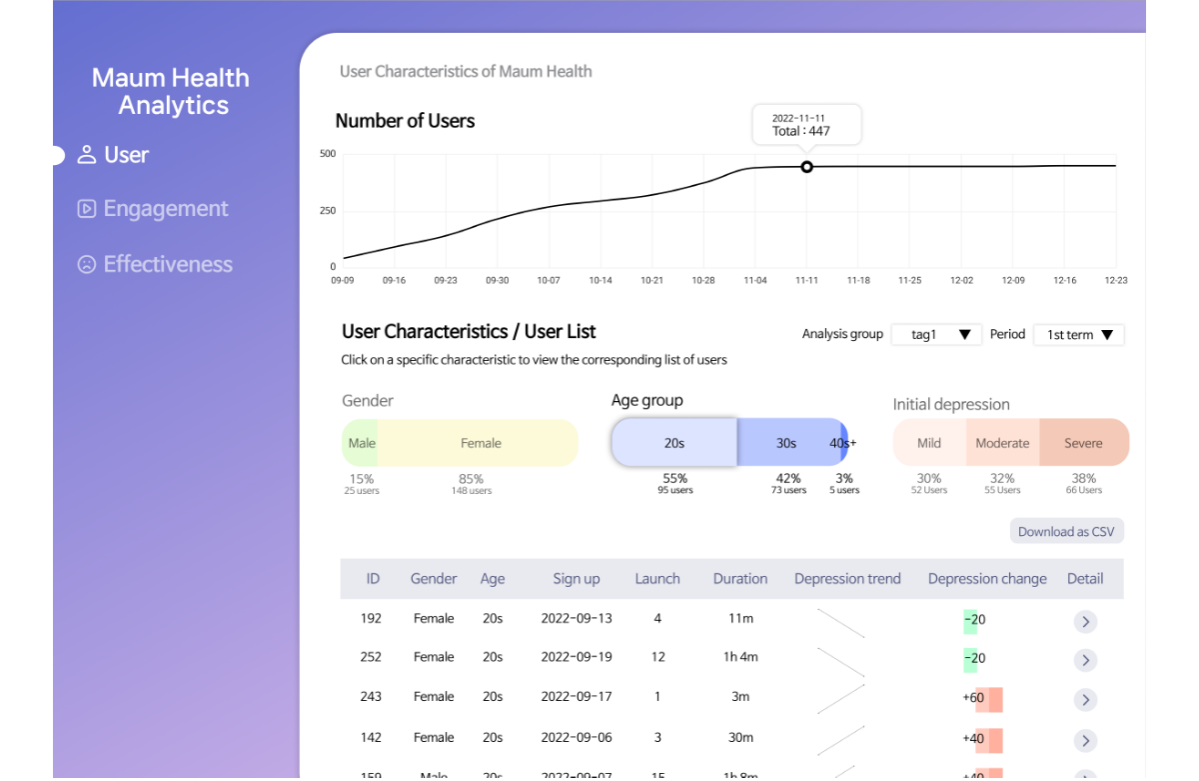
The User page of the Maum Health Analytics tool that supports tasks associated with user characteristics.

The User page (Figure 2) supports tasks associated with user characteristics, presenting visualizations of the number of DHI users over time (task T1.1) and the distribution of users by characteristics (task T1.2). Researchers can select specific user characteristics from the horizontal bar chart in the middle; users matching these criteria are displayed in the table that appears under User Characteristics and User List. Furthermore, they can select a specific user from the table to review detailed records (task T1.3), including the user’s basic information, engagement levels with DHIs, changes in depression levels, and individual DHI content use records.

Line charts were selected to effectively show temporal trends in user participation (task T1.1), whereas bar charts allowed for a clear comparison of categorical variables, such as age or gender (task T1.2). The use of a table enables detailed record-level exploration for individual users (task T1.3), thereby supporting granular analysis.

**Figure 3:**
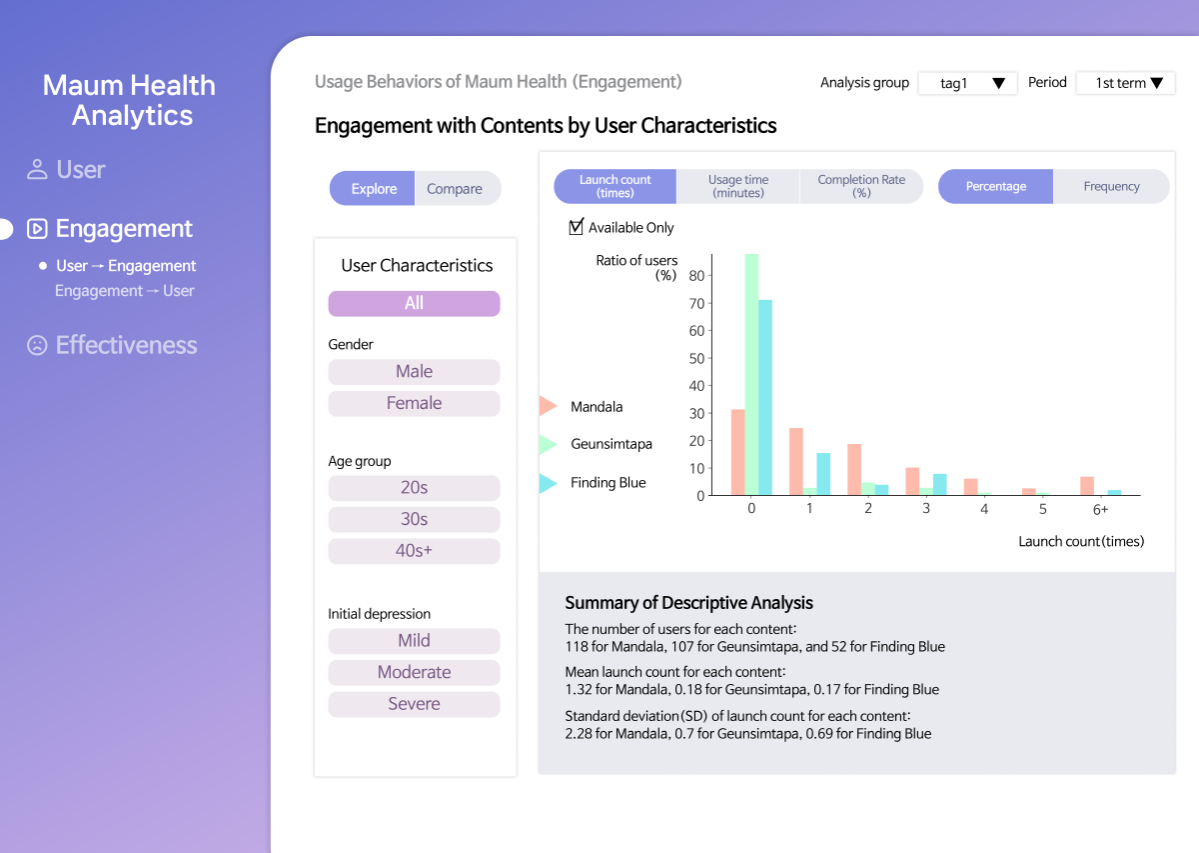
The Engagement page of the Maum Health Analytics tool that supports tasks associated with user engagement with digital health interventions (DHIs).

On the Engagement page (Figure 3), researchers can explore the engagement level of each DHI content item across all users (task T2.1) or within selected user groups (task T2.2) specified by user characteristics. As shown in the figure, the histogram illustrates the distribution of engagement levels within the selected user group, providing insights into the overall use behavior for each DHI content item. In addition, researchers are allowed to compare the engagement level across different user groups (task T2.3) to identify the users who are more engaged with each DHI content item. Furthermore, user groups can be formed based on specific engagement levels to examine the user characteristic distribution within these groups (task T2.4).

Histograms were used to visualize engagement distributions across user groups (tasks T2.1 to T2.3) as they effectively represented frequency patterns and enabled quick comparisons among subgroups. Bar charts were selected for task T2.4 to show how demographic characteristics vary by engagement level, thereby supporting the group-based analysis.

**Figure 4:**
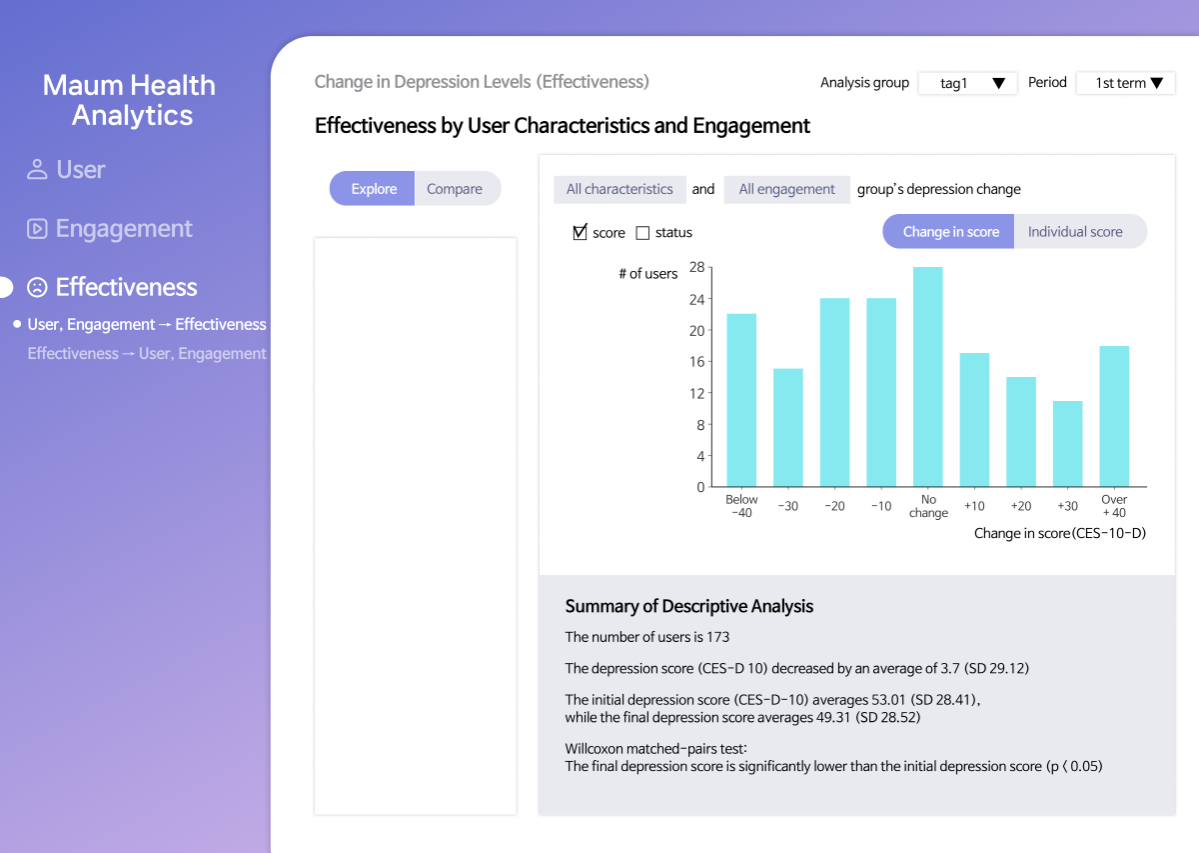
The Effectiveness page of the Maum Health Analytics tool that supports tasks associated with the effectiveness of digital health interventions (DHIs) on the target symptom.

Finally, researchers can explore the changes in depression levels on the Effectiveness page (Figure 4) either across all users (task T3.1) or within selected user groups (task T3.2). When specifying user groups for investigation, researchers are allowed to select user characteristics or user engagement with the DHI content, and the results are shown in a histogram. Similar to the Engagement page, researchers can compare changes in depression levels across different user groups (task T3.3). In addition, they can create user groups based on depression changes to understand the distribution of user characteristics and engagement levels within these groups (task T3.4).

Histograms were used to visualize changes in depressive symptoms before and after the intervention (task T3.1) as they are well suited for showing the distribution of continuous outcome variables and identifying general patterns of improvement or decline. For tasks T3.2 and T3.3, histograms were used to view or compare symptom change distributions across subgroups according to user characteristics or engagement, allowing for an intuitive visual comparison of group-level outcome variations. For task T3.4, bar charts were selected to summarize user characteristics and engagement within the effectiveness-based subgroups, thus supporting the identification of traits associated with greater or lesser effectiveness.

Further details about the Maum Health Analytics tool are provided in Multimedia Appendix 1.

### Preliminary User Study

#### Maum Health Dataset

As previously mentioned, we decided to provide the analysis results derived from the data collected through our DHI service, Maum Health, when evaluating the interactive visual analytics. Here, we provide a brief description of Maum Health and the data it collected.

#### Maum Health

Maum Health is a DHI service developed to improve depressive symptoms and is offered as a mobile app. Similar to typical mental health mobile apps, Maum Health provides various intervention content beneficial for depressive symptoms, including art therapy (Mandala), physical activity (Geunsimtapa), and a cognitive-emotional screening game (Finding Blue). Each intervention content item consists of sessions comprising activities that users can perform on their own. For instance, users can make color drawings, perform walking and stretching exercises, and play interactive games when engaging with each content item. Moreover, it assesses users’ depression levels every 2 weeks using the CES-D-10, a well-established and validated instrument for assessing depressive symptoms in mental health research. The results are converted to a 100-point scale, where a higher score indicates more severe depression.

#### Dataset

The Maum Health DHI service was distributed through a public counseling center located in Seoul, South Korea, which operates under the Seoul Metropolitan Government. The center is situated in an urban residential area with high accessibility via public transportation and provides mental health counseling services specifically for young adults, supported by licensed psychiatric professionals. As part of the counseling process, the center recommended Maum Health as a supplementary digital tool for managing mental health. Consequently, this DHI was used by 529 people over approximately 3 months, starting in September 2022. During this period, self-reported data entered by the users and log data automatically recorded based on the use of the intervention content were collected.

The self-reported data in Maum Health consisted of one-time basic user information and periodically recorded levels of depressive symptoms. When users first registered for Maum Health, they provided demographic information, such as gender and age, along with information that might be related to depressive symptoms (eg, marital status and alcohol or smoking experience). Moreover, while using Maum Health, users reported their depressive symptom levels biweekly through the CES-D-10 survey.

As users engaged with the 3 different types of intervention content available on Maum Health, log data were recorded automatically. Whenever a user finished a session with specific content, the time stamps for the start and end points of the session were recorded. Furthermore, for each session, the completion rate was recorded to indicate how well the user performed the given activity. We used features from both self-reported and log data in the design of the Maum Health Analytics tool, as summarized in Table 2.

**Table 2:** Features of the Maum Health DHI service data used in the design and evaluation of the Maum Health Analytics tool.

Analysis components and categories			Features
**User characteristics**			
	Basic information	Gender, age group, and initial depression level	
	Additional information	Marital status, cohabitant, occupation, education, economic status, drinking, smoking, army experiences, and handedness	
	Medical history	Depression history, medication, and physical illness	
**User engagement with DHIs^a^**			
	Mandala (art therapy)	Total launch counts, total use time, and average completion rate	
	Geunsimtapa (physical activity promotion)	Total launch counts, total use time, and average completion rate	
	Finding Blue (cognitive-emotional games)	Total launch counts, total use time, and average completion rate	
**Effectiveness of DHIs**			
	Depression level	CES-D-10^b^ score	

^a^DHI: digital health intervention.

^b^CES-D-10: 10-item Center of Epidemiologic Studies Depression Scale.

Considering the potential quality issues with RWD, we established inclusion and exclusion criteria for the data to be used when evaluating the Maum Health Analytics tool. We selected users who had at least 2 depression score records, which allowed us to observe changes in depression levels. Among them, we included users with a gap of 2 to 4 weeks between evaluations, as they were considered to maintain their DHI use sufficiently well. Consequently, we used data from 173 (32.7%) of the 529 users. Recognizing that the initial use time of the Maum Health DHI service could vary among users, we considered the relative use period starting with each user’s first day of use. As a result, the final dataset used to evaluate the Maum Health Analytics tool included 3470 data points, comprising user-provided demographic information, preprocessed log records of DHI content use, and periodic self-reports of depressive symptoms.

#### Study Procedure

##### Participants

To evaluate the Maum Health Analytics tool, we recruited 5 groups of experts with experience in analyzing DHI data. Each group comprised 3 to 5 experts, including clinicians, intervention content designers, and system developers, who collaborated as a team. In total, 15 DHI researchers participated in the study (woman researchers: n=6, 40% and man researchers: n=9, 60%), who were aged between 26 and 44 years (mean 34.1, SD 6.4 years). All participants had previous experience in developing DHIs and analyzing real-world DHI data. Although they had not used the Maum Health DHI service as end users, they became familiar with its structure and features through their involvement in related research and evaluation activities. The details of these expert groups are listed in Table 3, and we will refer to these experts as “DHI researchers” hereinafter.

**Table 3:** Composition of participants by digital health intervention research teams.

Group IDs	Participant IDs	Expertise of the group
A	A1, A2, and A3	Clinicians and intervention content designers
B	B1 and B2	Clinicians and intervention content designers
C	C1, C2, C3, C4, and C5	Intervention content designers
D	D1, D2, and D3	Clinicians, intervention content designers, and system developers
E	E1 and E2	System developers

##### Evaluation

Textbox 1 provides an overview of the evaluation procedure, comprising the research introduction, scenario-based use session, qualitative interviews, and quantitative usability assessment. During the user study, we first briefly introduced our research and explained the Maum Health Analytics tool, along with the dataset used for the evaluation, to the participants. Then, we asked them to explore the various features of our tool. Given that our tool was a medium-fidelity prototype, the participants were only able to navigate predefined functions, interactions, and corresponding analysis results.

**Overview of the study procedure for evaluating the Maum Health Analytics tool.**

**Research introduction**

- The authors introduced the study and provided an overview of the Maum Health Analytics tool.

**Scenario-based use session**

- Participants explored the Maum Health Analytics tool for 30 min.
- Realistic data analysis scenarios were provided to support system evaluation during this session.

**Qualitative evaluation**

- Semistructured interviews were conducted for 60 to 90 min.
- Participants evaluated the analysis tasks and discussed their applicability in real-world settings.

**Quantitative evaluation**

- The Post-Study System Usability Questionnaire was administered to assess the system’s usability.

In the evaluation session, we presented participants with specific DHI data analysis scenarios, enabling them to test all the tasks supported by Maum Health Analytics. For instance, if the scenario was “The expert investigates the distribution of user characteristics among those who frequently used Mandala above average,” the participants would follow several steps within the tool to analyze the data. Considering the limited functionality of the prototype, these scenarios were selected based on cases with sufficient Maum Health data to showcase the results completely. We visualized the analysis results from real-world DHI data to make the evaluation realistic, ensuring that the participants could interact with and assess the prototype in a meaningful context.

After the participants used the Maum Health Analytics tool, we conducted an interview session and asked key questions from the perspective of analyzing the Maum Health DHI service data: (1) Were the analysis tasks defined in our tool appropriate? (2) How could the information provided be used in practice? Each session lasted approximately 60 to 90 minutes, depending on the participant group’s pace and discussion depth. All interview sessions were recorded with the participants’ consent and transcribed to thoroughly examine their responses. Thereafter, we performed inductive analysis [45] while repeatedly reading the interview transcripts to identify key phrases, ideas, and themes. Next, we conducted affinity diagramming to group similar themes derived from the transcripts and reviewed the themes iteratively until all researchers agreed on the final themes.

We also used the Post-Study System Usability Questionnaire (PSSUQ) [46], which is based on a 7-point Likert scale, to evaluate the overall usability. The PSSUQ is specifically designed for scenario-based usability studies and includes a subscale for information quality, allowing us to quantitatively assess whether the defined analysis tasks are suitable. According to Lewis [47], the PSSUQ scores for real-world systems commonly ranged between 2.5 and 3.0 across subscales, with lower scores indicating better usability. Although no absolute benchmark has been proposed, scores within this range have been interpreted as indicating acceptable usability in practical settings. We used the Korean-translated version of the PSSUQ previously developed and validated by Jeon and Park [48]. The participants in our study did not report any difficulties in interpreting or responding to the translated version during the usability evaluation.

We analyzed the responses using descriptive statistics and computed the mean and SD for each of the 3 PSSUQ subscales: system usefulness, information quality, and interface quality. Two items related to error recovery (items 7 and 8) were excluded because the user scenarios used in this study did not involve any error-handling tasks.

### Ethical Considerations

This study was approved by the Institutional Review Board of the KAIST university (KH2022-098), and written informed consent was obtained from all participants.

## Results

### Overview

The previous section introduced the analysis task model and visual analytics tool developed for DHI research. On the basis of them, we conducted a preliminary user study to evaluate the usability of the system. This section presents the results of this evaluation, including both quantitative and qualitative findings.

### Quantitative Evaluation

The PSSUQ comprises 16 items across 3 subscales (ie, system usefulness, information quality, and interface quality) rated on a 7-point Likert scale (1=strongly agree, 4=neutral, 7=strongly disagree), with lower scores indicating better usability.

For the Maum Health Analytics tool, the mean score of the PSSUQ was 2.89 (SD 1.02), and that of the overall usability was 2.80 (SD 1.57). Each subscale had similar mean scores: system usefulness 2.94 (SD 1.01), information quality 2.87 (SD 1.21), and interface quality 2.84 (SD 1.31). These scores fell within the range commonly observed in previous usability studies (2.5-3.0) and are generally interpreted to reflect acceptable usability levels [47].

### Qualitative Evaluation

#### Analysis Task Model

Our interviews revealed participants’ opinions on the analysis task model and its components proposed in this study.

#### User Characteristics

Participants found that tasks related to user characteristics helped enhance their understanding of user groups, particularly those sharing specific characteristics. They were interested in examining individual use records as concrete examples of the findings identified at the group level. By reviewing representative users within a group, they can understand broader trends and verify their interpretations:

When I discovered that females used Mandala more, I became curious about each female user’s usage patterns. Reviewing their specific usage logs could help me identify common characteristics of this user group.

A1

The participants further noted that such an in-depth exploration was challenging in their previous practice, particularly when user interactions were recorded only on personal devices and were not easily accessible:

We could analyze DHI usage statistics by group, but it was challenging to check detailed usage logs since we had to take their phones for analysis.

A3

These comments highlighted that visualizing individual-level use data supported a deeper and more nuanced understanding of group-level patterns.

In addition, the participants reported that tasks in this category were useful for identifying DHI users (or user groups) who may require additional attention. They emphasized the importance of evaluating whether users met a minimum level of engagement (in launch counts or use time) within a given period and examining specific users who fell short of these thresholds:

For instance, if there is a weekly recommended use, I’d like to see how many users in the group meet that goal.

B1

The participants reviewed group-level engagement metrics and paid particular attention to individuals with low engagement within those groups. Among the participants, clinicians focused on improving users’ target symptoms and discussed how such insights could inform intervention decisions:

I was wondering why this user didn’t use Maum Health contents that much, and why her status got worse even while using intervention contents.

A1

They planned to intervene with users with low engagement to increase their engagement:

Perhaps I need to decide how to manage the low engagement users, for instance, send a text message, make a call, and so on.

B1

Some participants also mentioned that tagging could help monitor users who required closer attention:

In our study, there are individuals whom doctors have to care for more intensively. Previously, we had to find such users whenever we analyzed them. But here, I think it would be easier and more convenient because we can mark them with tags and monitor them accordingly.

C2

E1 viewed tags as customizable user characteristics and noted that using them as favorites made it easier to track certain users. Together, these findings suggested that being able to review individual user records within user characteristic tasks helped to identify users at both the group and individual levels who might require further intervention to improve their symptoms.

The participants also mentioned that tasks supported by Maum Health Analytics could help track new incoming users and monitor the retention of existing ones. They noted that understanding the characteristics of both groups was essential for delivering appropriate intervention content:

There is a difference between user groups by their retention rate. Based on that information, we could determine the frequency of exposing specific content for each user group, which might be available here, too.

D1

However, participants emphasized the need to distinguish “active users” from merely registered ones, identifying this as a potential area for improvement. For intervention content designers, knowing who actively engaged with the system was considered more important than simply tracking overall registrations:

I’d like to see how many users are actively using DHI, not just registered. For designers, it is important to see whether they are using DHIs well enough.

C1

To better determine active use, the participants suggested including session-level dropout information on individual user pages:

I think the quitting ratio while using content is also important. The intervention content may be interesting at the beginning, but it gets boring as time goes

C3

These findings implied that supporting both retention tracking and active user identification contributed to a comprehensive understanding of DHI users and facilitated effective user management planning.

#### User Engagement With DHIs

Participants reported that Maum Health Analytics could help them identify user groups that exhibited high engagement with specific DHI content, serving as useful information for making more effective content recommendations. Tasks related to user engagement, such as task T2.4, provided a distribution of user characteristics among highly engaged users. For instance, when extracting users who used Mandala above the average level, participants observed an increased proportion of woman users, which they found helpful in decision-making:

After examining the characteristics of users who use a particular content often, we can recommend it to similar users, such as those in the same age group. Maybe we can promote the DHI service by targeting similar groups of people through social media.

A1

The participants assumed that users with similar characteristics would demonstrate similar engagement levels. They considered this assumption valuable for improving recommendation strategies for specific groups of users, particularly because DHI content is typically recommended based solely on target symptom severity (eg, depression scores) without considering user preferences or engagement patterns:

I think this tool can be used to make feedback more customized while monitoring the changes in DHI engagement.

A2

Conversely, some participants indicated that users with low engagement should be further examined to design new content tailored to them. For instance, a participant noted the following:

If the proportion of females in users with high engagement is significant, we should create another content targeting men. Otherwise, the male user group may not be supported well and might leave the Maum Health app.

A3

However, B1 noted that recommendations based solely on engagement should be used cautiously and that the actual improvement in depressive symptoms should also be considered. These findings demonstrated that examining engagement patterns in conjunction with user characteristics can support more personalized and effective content recommendations while also highlighting the importance of considering intervention effectiveness alongside DHI use behavior.

Some participants mentioned that presenting the overall distribution of engagement with the intervention content was useful, as it could help establish guidelines for DHI use. They noted that interventions such as traditional cognitive behavioral therapy can be easily evaluated in terms of engagement because the number of sessions is predefined. However, setting evaluation criteria is challenging when users use the content independently without specific guidelines.

After exploring the overall engagement levels and those of specific user groups through the Maum Health Analytics tool, participants expected to establish engagement guidelines using this tool as more DHI data accumulated over time:

If the data becomes sufficient, values such as average or top 25% could be more meaningful. Observing the overall engagement level with this tool, we can provide engagement guidelines for each step or set a recommended use.

B1

This indicated the potential for establishing optimal use guidelines by analyzing long-term DHI engagement patterns, which currently lack established benchmarks.

In addition, the participants mentioned the need to evaluate user engagement more rigorously. In particular, the system developer group emphasized the importance of selecting appropriate use metrics, such as launch counts and use time, when measuring engagement. Compared with other groups, they primarily focused on improving usability and maintaining overall engagement levels rather than evaluating the effectiveness of DHIs. Consequently, they prioritized analyzing detailed content use logs and identifying metrics that more accurately reflected the actual user behavior:

For example, there are cases when users stop using content when getting a call or eating. In those cases, they stayed in the content but there was no interaction recorded in the usage log.

E1

The participants also highlighted the importance of distinguishing what constitutes “effective” engagement, thereby emphasizing the need to focus on use behaviors that indicate meaningful interactions with content:

It is necessary to analyze how much users actively participated in the content before investigating its effectiveness. Then, we have to further examine what was behind if DHI turned out to be ineffective.

E2

This implied that measuring engagement in DHIs should go beyond basic metrics, such as launch counts or use time, and instead incorporate indicators that more accurately capture users’ actual interaction behaviors.

#### Effectiveness of DHIs

Participants responded that the Maum Health Analytics tool was helpful in systematically evaluating the effectiveness of DHIs by examining changes in target symptoms. Because DHIs are intended to support users’ mental health, the participants found it meaningful to analyze symptom improvement in relation to engagement with each type of intervention content:

Of course, showing individual indicators can be a meaningful analysis for almost all apps. However, for DHIs, we should investigate which intervention features interact with each other and how they improve symptoms, requiring a relational analysis of multiple factors.

D1

For example, for game apps, the longer the usage time, the better. But in the case of DHIs, we need to see not only how long it has been used, but also how effective it is. I think this tool supports this process well enough.

E1

The participants compared Google Analytics with our tool, highlighting a key difference in their purposes. They explained that the key distinction of the proposed analytics tool was the inclusion of health-related indicators, making it more suitable for analyzing DHI services:

Unlike Google Analytics, which mainly focuses on usage behavior and user retention, we can analyze DHI including the users’ health indicators in this tool. This is critical for health-related services.

D1

This suggested that evaluating the effectiveness of DHIs required integrating health outcomes with use metrics and that Maum Health Analytics enabled such multidimensional analysis more effectively than existing analysis practices.

We observed that evaluating content effectiveness from multiple perspectives using Maum Health Analytics was considered beneficial by participants, particularly in the context of designing or studying the intervention content. They explained that the tool simplified comparisons that would otherwise require repeated statistical analyses and manual setup under each condition:

Previously, it was inconvenient to repeatedly perform statistical analysis for each condition, so we sometimes skipped examining some questions in detail. Perhaps, we lost opportunities to find something new.

C1

By supporting comparisons based on user characteristics and engagement levels, the tool enabled users to explore effectiveness across different subgroups more efficiently:

Like the analysis in research, I was able to create control groups and experimental groups based on several criteria and easily compare the difference in depression change between the groups. I could see the relationship between engagement and effectiveness through this process.

C2

The participants also highlighted the tag feature as a practical way to track and compare user-defined groups. This allowed them to monitor symptom changes in relation to factors such as the app version or intervention updates:

I like the tag because we can immediately see how much DHI has improved after updates if we label tags based on the version of DHI.

C1

C1 added that using tags to compare groups could help the research team formulate and evaluate new hypotheses, similar to A/B testing.

In addition, participants found it useful to evaluate other indicators based on the change in depression levels. As DHI services are still in their early stages, no clear evidence exists that increased use leads to better outcomes. Therefore, B1 suggested that it might be more insightful to investigate other indicators based on the effectiveness of the DHI. These findings suggested that enabling flexible group comparisons and supporting exploratory evaluations of DHI effectiveness can help researchers uncover new patterns, generate hypotheses, and refine intervention strategies based on subgroup insights.

#### Interactive Visual Analytics

We also explored participants’ experiences with the interactive visual analytics tool during the interview sessions.

#### Simplifying the Analysis Tasks of DHI

Participants mentioned that using the Maum Health Analytics tool helped simplify several of the manual and repetitive tasks they typically faced in DHI data analysis. In particular, they appreciated not having to download the entire dataset each time, recognizing this as one of the most practical features:

Our lab is developing a similar DHI app, but we have to download and analyze the entire dataset since there’s no analytics tool like this. But here, we can check what happened to the user right away without such tasks.

A3

Because most existing dashboards were built for experiment management instead of analysis, participants found it helpful that our tool efficiently supported basic analytical tasks.

Participants also appreciated the ability to view analysis results in real time:

I have experience in developing chatbot-based interventions, and I wanted to know in real-time whether users are using them well or how they respond. I was satisfied to see that information on this tool.

A3

Real-time information may be meaningful to those who provide counseling services based on analyzed results, those who manage certain groups of users, and those who monitor the entire service.

B1

The participants also appreciated that the tool allowed them to extract and export user groups for further analysis. In particular, A2 and A3 highlighted the usefulness of isolating specific subgroups, such as outliers with unusual engagement or symptom patterns, and downloading their data to examine them in more detail using external analytical tools. These findings highlighted how Maum Health Analytics can reduce the burden of manual data handling and facilitate a more efficient and targeted analysis workflow.

#### Supporting Communication Among Various Experts

The participants assessed that the Maum Health Analytics tool would facilitate communication among stakeholders during the DHI improvement process, particularly when experts from different domains collaborate. They emphasized that the tool could bridge the gaps between those with differing levels of expertise in data analysis and interpretation.

Previously, content designers had to create separate documents to explain analysis results to other domain experts, which they found to be inefficient:

As a content designer, I always had to prepare additional materials about the analysis results to explain it to mental health counselors. However, with these visual explanations, I think they can understand the results easily without any further documents.

A3

They added that even stakeholders unfamiliar with data analysis could interpret the visual results and answer relevant questions using Maum Health Analytics:

Since this tool showed the results visually, I thought it would be suitable for those who are not familiar with how to analyze data well. With this visual analytics tool, they would be able to answer various questions related to DHI usage.

A2

Participants also noted that in cases where data analysis and user recruitment were handled by separate individuals or teams, the tool could help track and understand the user base:

If someone else is recruiting DHI users, there will be difficulties in the analysis since we have to examine the data without understanding the participants. This tool displays that information right away, making it easy to track DHI users’ information even if we don’t recruit the participants by ourselves.

C1

These findings suggested that Maum Health Analytics could serve as a shared platform to enhance communication and understanding among DHI stakeholders with diverse roles and technical backgrounds.

#### Providing Statistical Analysis and Interpretation of Results

The participants reported that the Maum Health Analytics tool was useful in providing statistical analysis results when evaluating engagement and effectiveness. They emphasized that statistical testing added an objective basis for interpreting and presenting findings:

There must be people who think statistics are important. For example, in the public domain of managing these studies, it is important to leave a numerical rationale.

B1

If we say that this result is statistically significant, I think we can clearly communicate it to experts in other fields.

C3

The statistical analysis was meaningful, particularly because most of the results were presented visually in graphs. However, the participants suggested that these results should be accompanied by more insightful explanations to better support DHI improvement. Although descriptive statistics, such as means and SDs, can be roughly interpreted from visualizations, some participants noted that providing statistical testing results along with practical interpretations would be more beneficial:

When considering people who are not familiar with statistics may see statistical analysis, it would be nice to provide interpretations related to decision-making, such as what this result means and how to utilize it in updating DHIs.

C1

These findings indicated that providing statistical results together with clear, practical interpretations could improve the usability of analytical tools and help stakeholders better understand and apply insights.

#### Enhancing Continuity Across Analysis Tasks

Participants commented that the transition between different analysis tasks could be improved by allowing the currently selected user groups to be pinned. They expressed the need to fix a group of interests and view all subsequent analysis results for that group:

For example, if I went to another page after checking the engagement distribution of Mandala, I wanted to view the analysis results of the same user group first.

B1

One participant specifically emphasized the need for a feature that allowed saving filtered user groups:

We first narrowed the user group down by adding more filters, but we had to find and select the conditions again if we moved to other pages. As a result, the analysis tasks seemed somewhat disconnected.

E1

They suggested that the ability to save and pin a specified user group could serve as a bridge between analysis tasks, enabling a more seamless experience when working with DHI data. They expected this feature to help researchers maintain continuity across tasks and pages by preserving their focus on the same user group. This implies that ensuring continuity between the analysis steps is a critical design consideration for supporting exploratory workflows in DHI data analysis.

## Discussion

### Principal Findings

#### Overview

We proposed an analysis task model for practitioners to explore and analyze RWD generated by DHIs. Building on previous studies related to DHIs, we developed a task model comprising key components, including user characteristics, user engagement with DHIs, and the effectiveness of DHIs. The model comprised data analysis tasks that explored not only each component individually but also their interrelationships. In addition, we designed an interactive visual analytics tool based on the proposed model to investigate how the model and tool could support diverse DHI data analysis practices. To make the evaluation more realistic, we visualized the analysis results from RWD collected from Maum Health, a currently operational DHI service, via the prototype and examined the DHI researchers’ user experience while using it.

Overall, the participants responded positively to the proposed model and the visual analytics tool. They noted that this supported analyses that were previously difficult or time consuming. Features such as reviewing individual user records, examining engagement and effectiveness from multiple perspectives, and comparing user-defined subgroups were helpful in improving the design and evaluation of DHIs. This evaluation also revealed several areas for improvement. The participants suggested including more detailed engagement metrics that reflected actual user interactions, adding functionality to retain selected user groups across views, and providing clearer interpretations of statistical results to support collaboration among researchers with different levels of data literacy.

In subsequent sections, we provide several insights into the analysis task model and the interactive visual analytics tool and suggest design implications for supporting researchers when analyzing DHI data.

#### Analysis Task Model

The analysis task model allowed the DHI research team to analyze the data holistically, addressing the limitations of existing studies that analyzed each key component separately. The proposed model supported the analysis of the relationships between key components by specifying user groups based on certain conditions (eg, gender) and comparing target outcomes (eg, changes in depression). During this process, the research team identified meaningful insights for improving DHIs, such as suggesting content to potential target users, setting criteria for DHI use, and determining better interventions. This aligns with recent studies investigating which DHI use behaviors (ie, engagement with DHIs) affect target outcomes [8,49], highlighting the importance of further analyses of these relationships.

In real-world settings, DHIs can be distributed through app stores to individuals with varying characteristics, contexts of use, and engagement levels [50]. Therefore, as suggested by our model, evaluating the DHI use behaviors and changes in target symptoms for each group of similar users is critical. Group-level analysis has been widely used in previous studies on large-scale health data [13,24,51]. These studies explored the overall trends or patterns at the user group level and derived insights to address common issues within the group. Given that DHI data can be collected from several users, DHI researchers may follow similar analytical practices.

In addition, the records of individual users can be provided as sample cases to complement the understanding of a specific user group. Because it is challenging to review all records, the analysis task model can be improved to suggest the representative users of that group. For instance, they could be users showing mean or median engagement levels or those with the most frequently reported user characteristics. This approach helps researchers gain a more nuanced understanding of user behavior and outcomes, allowing for more tailored and effective interventions.

Considering the researchers’ feedback regarding our prototype that they would set guidelines after exploring the engagement levels for each user group, group-level analysis could also be used to determine the optimal dosage. In practice, determining the dosage of typical DHIs is difficult because of various behavioral and contextual factors influencing dose-response relationships [14,16]. The proposed model could support researchers in investigating how much DHI use is required to achieve a certain level of improvement in the target symptoms. On the basis of this insight, they can further improve existing DHIs by adjusting the dosage or revising the difficulty of tasks to be performed in the intervention content while monitoring how these changes affect the effectiveness of DHIs.

#### Interactive Visual Analytics

The interactive visual analytics for DHIs proposed in our study demonstrated the potential of allowing researchers to perform analysis tasks efficiently. The findings of this study revealed that researchers often conducted similar DHI data analyses; however, there is a lack of research on visual analytics tools to streamline this process. The Maum Health Analytics tool has enabled researchers to analyze DHI data from diverse perspectives in real time through simple interactions.

The proposed analytics can be used at various stages of the development and evaluation life cycle of DHI services, which typically follow an agile software development process [23]. This process involves several stages of design and testing, with results from each testing fed back into the previous cycle to continuously improve DHIs. Therefore, researchers need to monitor and evaluate DHI data, and our tool can make this iterative process more efficient.

We also discovered that interactive visual analytics can be useful for DHI research teams comprising experts from diverse domains and backgrounds, such as clinicians, intervention designers, and system developers. As shown in this study, the purpose of data analysis may vary owing to background differences. For instance, clinicians value whether target symptoms have improved, whereas system developers focus more on the interaction log generated from DHI.

In such situations, the analyses provided by our analytics tool allow researchers to explore unfamiliar aspects of DHI data and extend their knowledge of the data. This facilitates communication among researchers from different fields, generating diverse approaches to better support users and improve existing intervention components. Furthermore, the analytical tool can be used to quickly share results with stakeholders outside the research team, such as funding bodies or external project managers, thus aiding the decision-making process.

#### Design Implications

Our findings offer further ways to quantify DHI use behaviors and diversify the metrics used to represent user engagement with interventions. As reported in this study, some DHI users may simply run the intervention content without actively engaging with it. This implies that relying solely on traditional high-level engagement metrics, such as launch counts or use time, may not distinguish this type of passive use, which could negatively affect the analysis quality.

Therefore, we propose incorporating fine-grained engagement metrics to better understand how active users interact with DHIs. For instance, rather than simply measuring how often a DHI is launched or how long it is used, we could track specific actions, such as touches, swipes, or clicks, within the app to obtain a clearer picture of user engagement [12]. Moreover, if the DHI includes activities that users are supposed to perform, the system could track their adherence to these activities and incorporate these data into the engagement metrics. By doing so, we can identify more meaningful (or effective) engagement and analyze its impact on improving target symptoms. This approach provides a more nuanced understanding of user behavior and the effectiveness of interventions.

In addition, we suggest design implications of interactive visual analytics tools for DHI researchers. Given that multiple user groups can be formed from data, determining the groups that should be investigated is challenging. Therefore, this tool can be designed to proactively identify the important user groups that should be examined by researchers. For instance, an analytics tool can recommend user groups with very high engagement or very low effectiveness, as these may provide critical insights. Leveraging the data analysis history stored in the analytics tool can provide analysis results that researchers are interested in or guide them to discover other important user groups.

Furthermore, we can support user group selection using natural language, making it easier for researchers to locate the groups they want to explore. This could be a practical alternative, particularly if numerous metrics are available for analyzing user engagement with DHIs and their effectiveness. In addition, enabling researchers to explore various factors based on a specified user group (ie, the tag function in this study) would enhance the user experience of visual analytics by allowing for seamless DHI data analysis.

When designing a visual analytics tool, both the exploratory and explanatory aspects should be carefully considered. Members of a multidisciplinary research team may have varying levels of knowledge and experience in data analysis. Therefore, interpreting the results is crucial for applying data-driven insights in practice. For instance, our tool can be further improved by explaining the statistical results and offering practical guidelines based on them.

With recent advances in large language models, visual analytics can generate reports based on data analysis histories thus enhancing researchers’ understanding of DHI data [52]. As suggested in a recent study [53], the data presented in visual elements can also be accompanied by a data-driven narrative generated by large language models, which effectively aids data communication. By integrating these features, the visual analytics tool can become a powerful and user-friendly resource for multidisciplinary research teams, facilitating deeper insights and more effective decision-making in the development and evaluation of DHIs.

### Limitations and Future Works

We conducted a preliminary user study on the interactive visual analytics tool designed based on the findings from our research experience and previous studies. The analytics tool presented in this study was a preliminary medium-fidelity prototype, with limited interaction capabilities intended for exploratory evaluation. In future studies, it will be necessary to develop a fully functional system based on this prototype and compare it with existing analytical tools. During this process, it is important to include DHI data collected over extended periods to allow users to evaluate how engagement and effectiveness evolve over time, as frequently observed in real-world DHI implementation.

Investigating how this analytics tool and the underlying analysis task model are applied in real-world settings is another important direction for future research, particularly for supporting DHI research teams more effectively. By addressing these aspects, we aim to create comprehensive and robust analytics that contribute to the field of DHIs.

In addition, the analytic task model and visual analytics tool proposed in this study were developed based on data from a mobile DHI service focused on mental health, specifically targeting depressive symptoms. Thus, the scope and tasks of the model may be influenced by characteristics unique to this domain. When applying the model to DHIs in other health domains, it is necessary to adapt the analytical components to reflect domain-specific requirements. For instance, physical and metabolic health interventions may rely on objective indicators, such as step count, blood glucose levels, or sleep duration, which require different approaches for modeling and evaluation. Similarly, engagement in these domains may involve different types of intervention activities and their corresponding completion metrics. In such cases, an analytical framework is needed to accommodate more nuanced metrics that better reflect user engagement and health outcomes. Furthermore, interventions involving real-time tracking or context-aware features may produce logs that require different forms of temporal or event-based analyses. Expanding the model to support such flexible representations and metrics is critical to enhance its applicability across diverse types of DHIs.

### Conclusions

We proposed an analysis task model to support DHI researchers by incorporating analysis practices from existing DHI research. On the basis of this model, we designed a prototype of an interactive visual analytics tool to explore how the proposed task model and tool can aid in DHI data analysis tasks. A preliminary user study demonstrated that the proposed model enabled a holistic investigation of DHI data by integrating the 3 key components and examining their relationships. Moreover, the analytics tool demonstrated the potential to simplify repetitive tasks and facilitate communication among researchers from diverse backgrounds and interests.

As the use of DHIs continues to grow, research on enhancing data analysis and supporting decision-making for improving existing DHIs has become increasingly important. In this context, we expect that the model and tool proposed in our study will effectively meet the data analysis needs of DHI researchers and make the process more efficient.

## Appendix

Multimedia Appendix 1 Description of the analysis tasks supported by the Maum Health Analytics tool.

## Abbreviations

CES-D-10: 10-item Center of Epidemiologic Studies Depression Scale
DHI: digital health intervention
PSSUQ: Post-Study System Usability Questionnaire
RWD: real-world data
