# Supplementary material for: Designing a Visual Analytics Tool to Support Data Analysis Tasks of Digital Mental Health Interventions: Case Study
Source: JMIR Hum Factors. 2025 Jul 2;12:e64967. doi: 10.2196/64967 (PMC12268220; doi:10.2196/64967)
Supplement: Multimedia Appendix 1 [file humanfactors_v12i1e64967_app1.docx]

# Multimedia Appendix 1

This Multimedia Appendix illustrates Maum Health Analytics, the interactive visual analytics tool built upon the analysis task model we proposed for DHI research.

Maum Health Analytics consists of three main pages: User, Engagement, and Effectiveness, each mapped one-to-one with the three key components of the analysis task model (i.e., user characteristics, user engagement with DHIs, and effectiveness of DHIs). The analytics tool is designed to allow researchers to perform a set of sub-tasks of the model on each corresponding page.

Below, we explain the detailed functions of Maum Health Analytics and how analysis results are presented for each page.

## User

The ‘User’ page enables the DHI research team to overview the information of Maum Health users (Analysis tasks T1-1, T1-2, and T1-3).

### User: Main Page


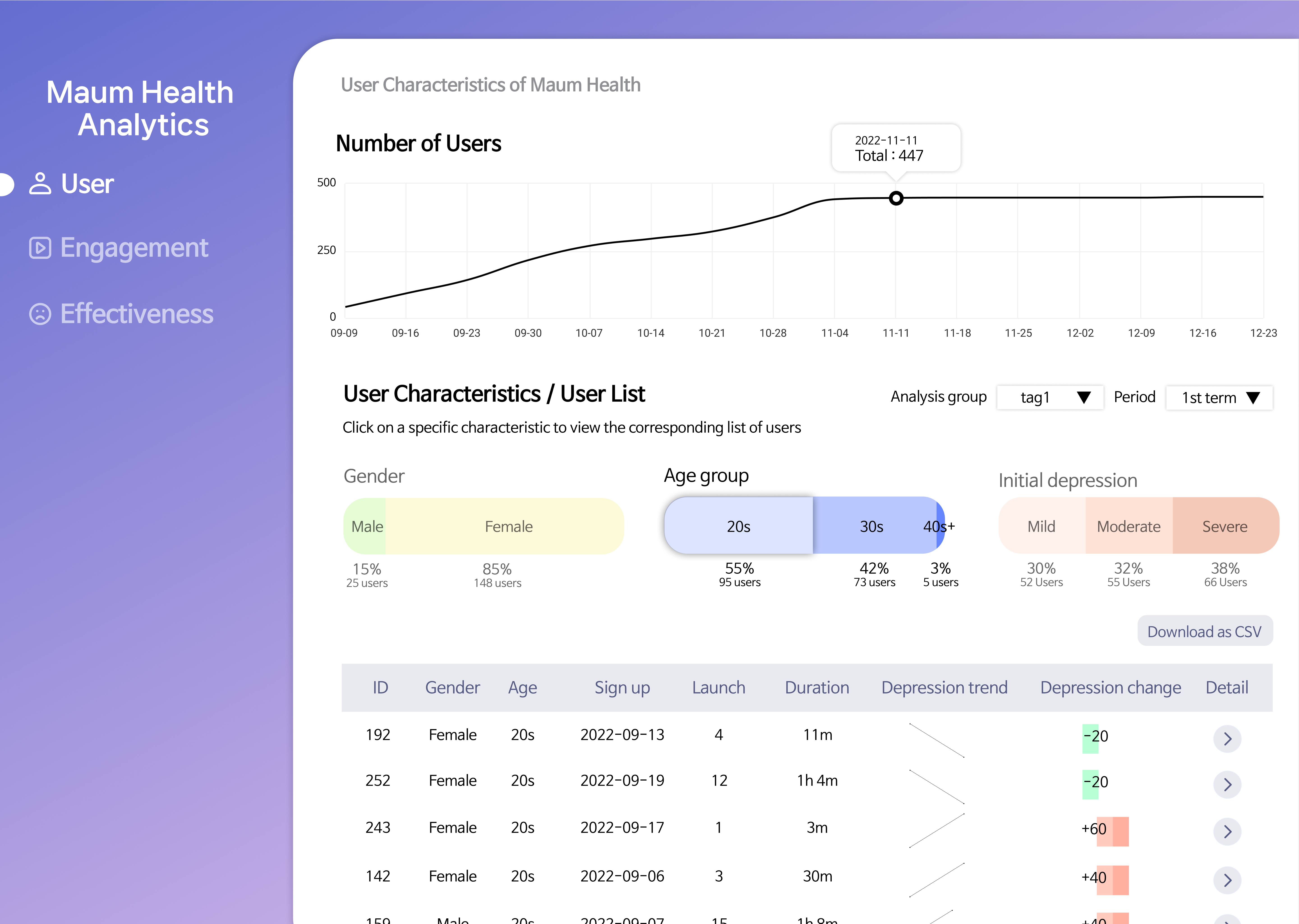


Figure 1. “User” page, displaying the number of users, the distribution of user characteristics, and the list of users

As illustrated in Figure 1, it presents the number of enrolled users over time, allowing researchers to review how many users are using the DHI. By clicking on a specific point on the graph, they can check the enrolled users as of that date. It then illustrates the distribution of Maum Health users by user characteristics, including gender, age, and initial depression state. The user distribution is visualized using bar charts, along with the number and proportion of users for each characteristic.

In addition, this page provides a list of users in a tabular format, where each row corresponds to a brief overview of an individual user. When researchers click on one of the user characteristics in the bar chart, the list of users is updated to show the corresponding users. For example, if they click '20s' on the user characteristic distribution chart, it works as a filter, displaying only the users in their twenties on the user list. Moreover, the extracted users based on certain user characteristics can be exported as a separate CSV file, allowing researchers to analyze the data further using other tools.

### User: Individual User Page


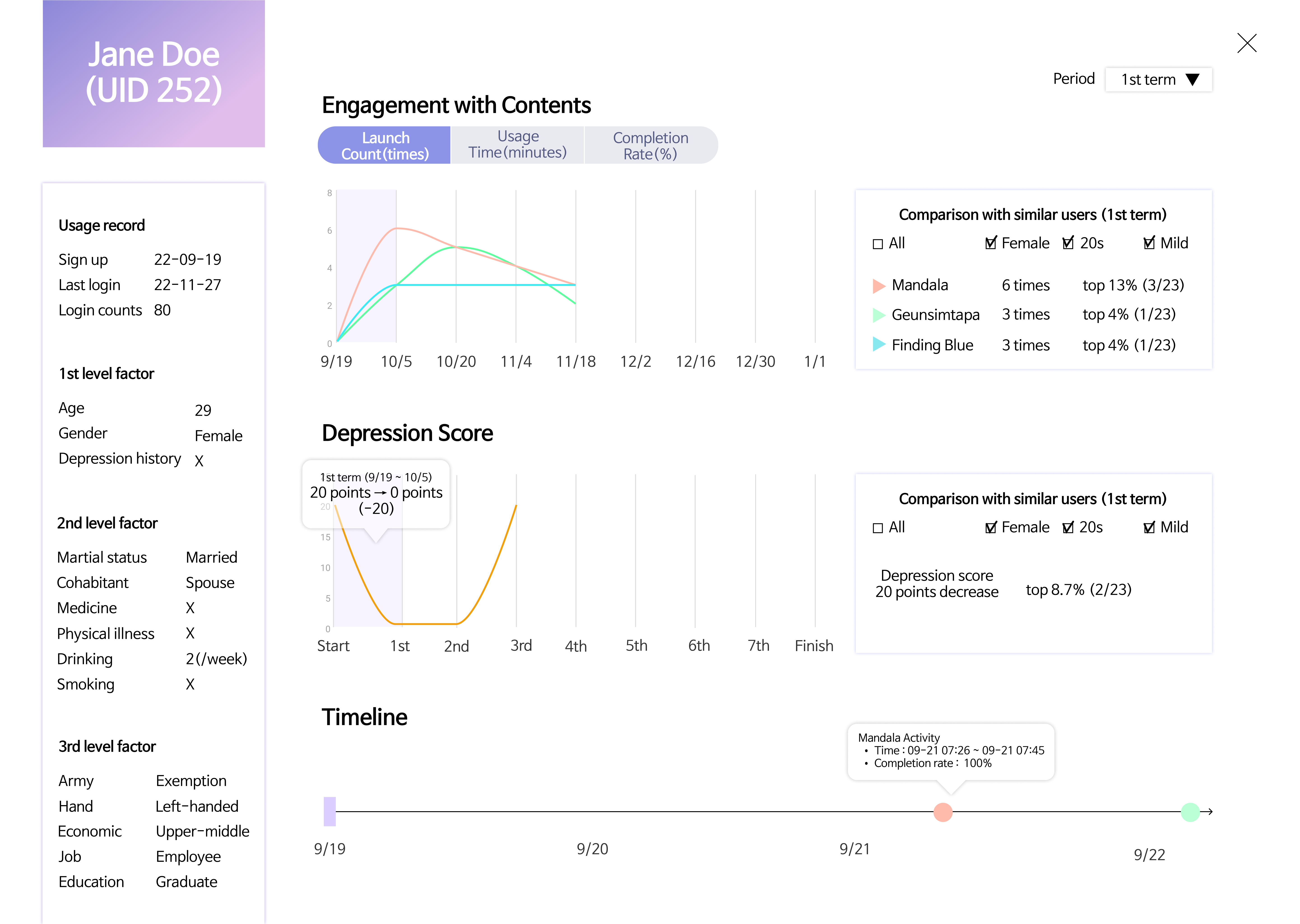


Figure 2. Individual user page, accessed by selecting a specific user from the user list in the “User” page

In the user list, DHI researchers can select a specific individual user and move to a sub-page showing the detailed records of that user. First, this page provides additional information about the user, which might be associated with symptoms of depression. The main section presents the user's engagement level with Maum Health content and depression level over time. Also, it explains the user's relative engagement and depression levels compared to other similar users.

As illustrated in Figure 2, researchers can investigate this user in comparison with users sharing the same characteristics (e.g., top 13% in Mandala launch counts among users who are female, in their 20s, and have a severe initial depression state). Furthermore, this page shows details of each intervention session and outcomes from the activity in a timeline. By exploring the information provided on this page, researchers can use these as sample cases to gain a deeper understanding of the user group.

## Engagement

The 'Engagement' page supports the DHI research team in examining user engagement with intervention contents in Maum Health (Analysis tasks T2-1, T2-2, T2-3, and T2-4).

### User characteristics → User engagement with Maum Health contents

#### Explore


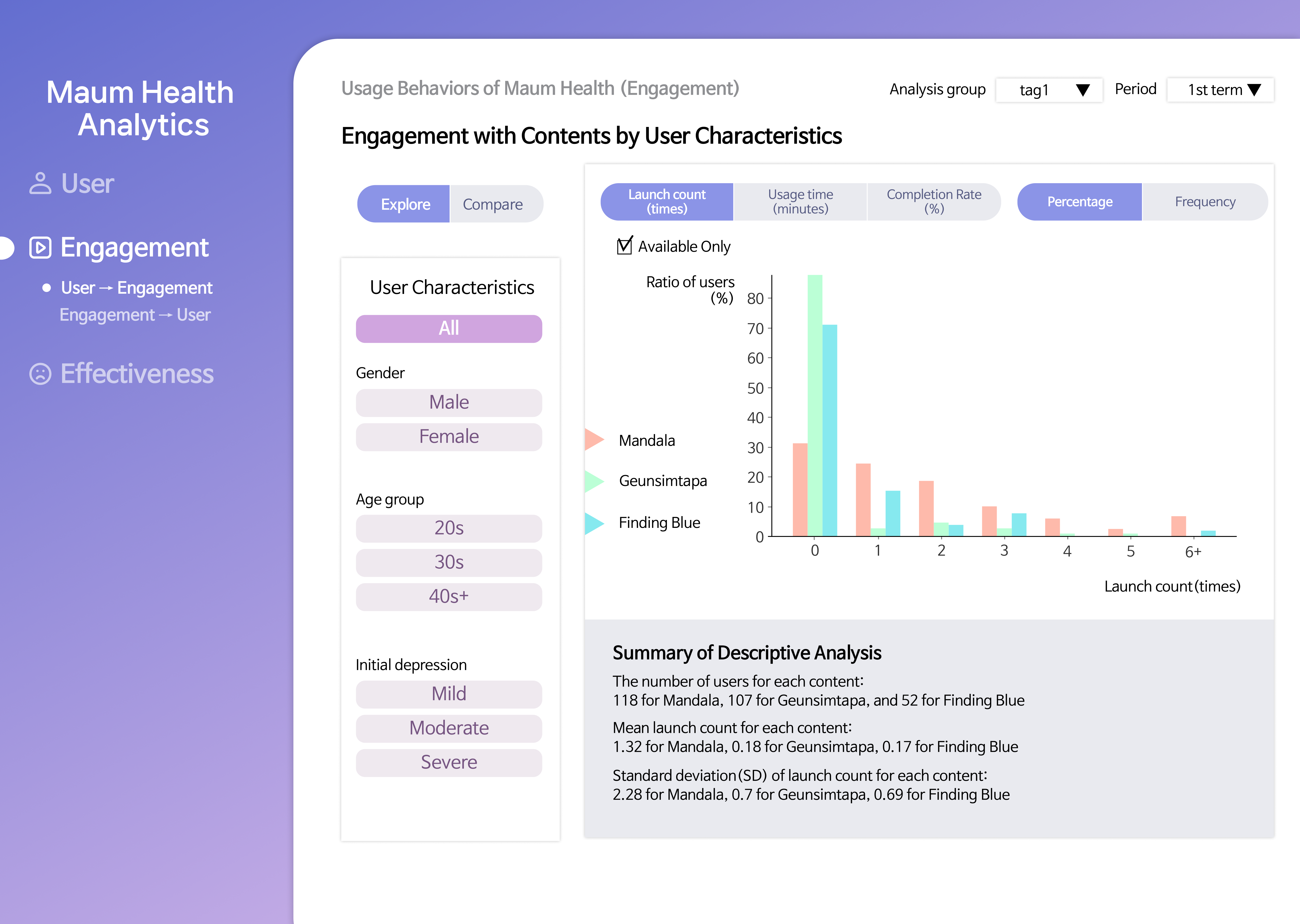


Figure 3. “Engagement” page, exploring the distribution of user engagement metrics for a user group specified by user characteristics

First, the 'Explore' menu allows researchers to examine engagement levels for various user groups. By default, this page presents the distribution of all users by engagement level for each intervention content. However, researchers can select a group of users sharing the same characteristics from the left panel to examine their engagement levels. Each user characteristic has unique values. For instance, researchers can investigate the engagement levels of female users by selecting the female option in the gender type.

As displayed in Figure 3, the page shows the distribution of users (in ratio) by their launch counts for Maum Health intervention contents. In this example, the bar chart shows that most users did not run the given intervention contents at all, and Mandala (the art therapy content) was used more frequently compared to other contents. Researchers can explore other engagement metrics, including usage time and completion rate, and view the actual number of users instead of their ratios. Moreover, descriptive statistics such as the mean and standard deviation of the engagement metrics for each content are provided below the chart.

In addition, since Maum Health recommended intervention content based on the user's recent depression state, the available content may have varied from user to user. Therefore, we provide an option for the chart, 'available only,' to distinguish between non-use and non-available cases.

#### Compare


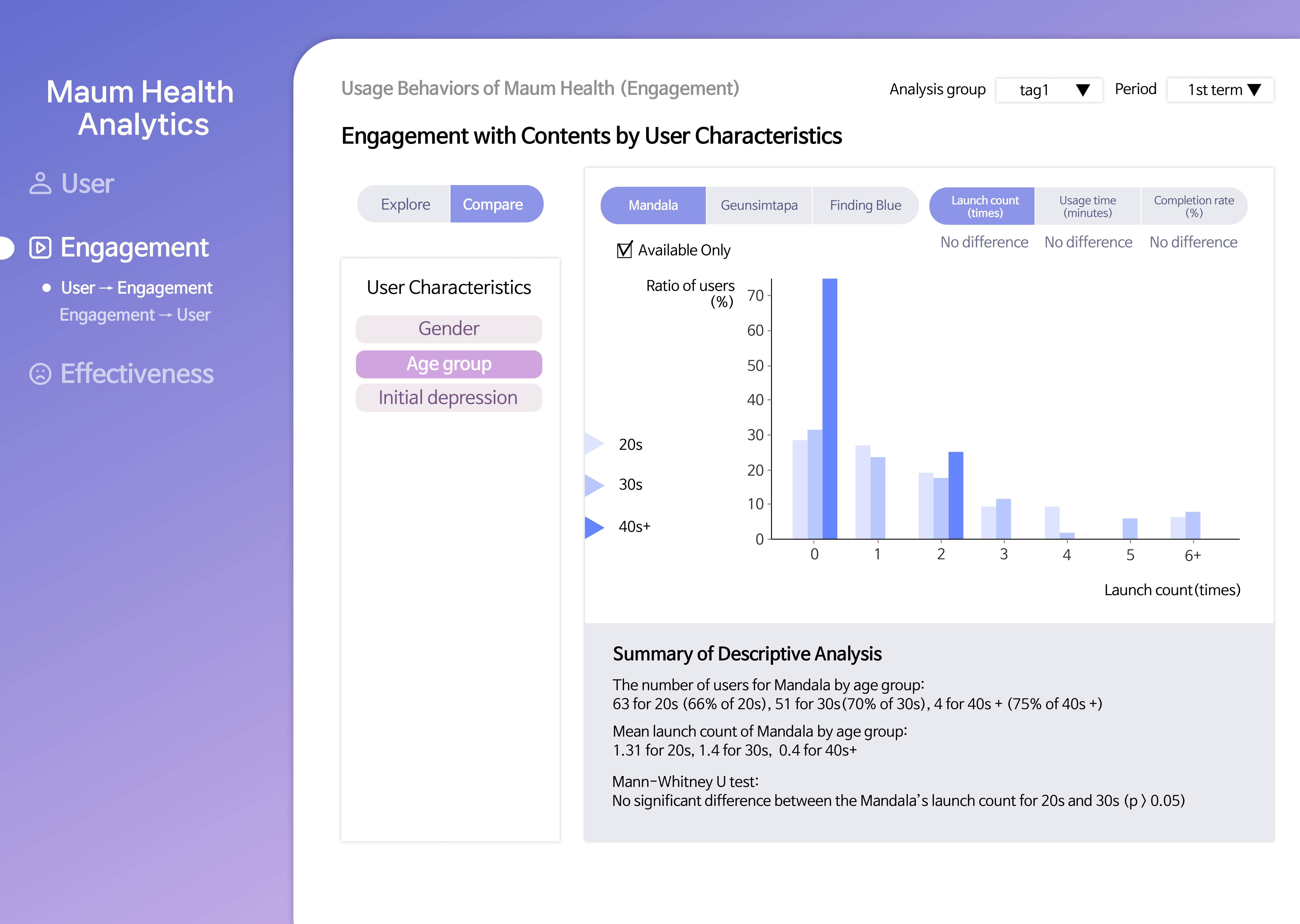


Figure 4. “Engagement” page, comparing the distribution of user engagement metrics between user groups specified by user characteristics

The 'Compare' menu enables researchers to compare engagement levels across user groups specified based on user characteristics. For example, if researchers select the 'age' category in the left panel, this page provides a bar chart showing the user distribution based on the launch counts of the Mandala content for users in their 20s, 30s, and 40s (Figure 4). Researchers can change the engagement metrics to usage time or completion rate and investigate other intervention contents as well. This page offers descriptive statistics for the selected engagement metrics and provides the results of statistical tests to determine whether there is a significant difference between user groups. Based on this information, researchers can identify which user groups need to be managed for active use of Maum Health.

### User engagement with Maum Health contents → User characteristics


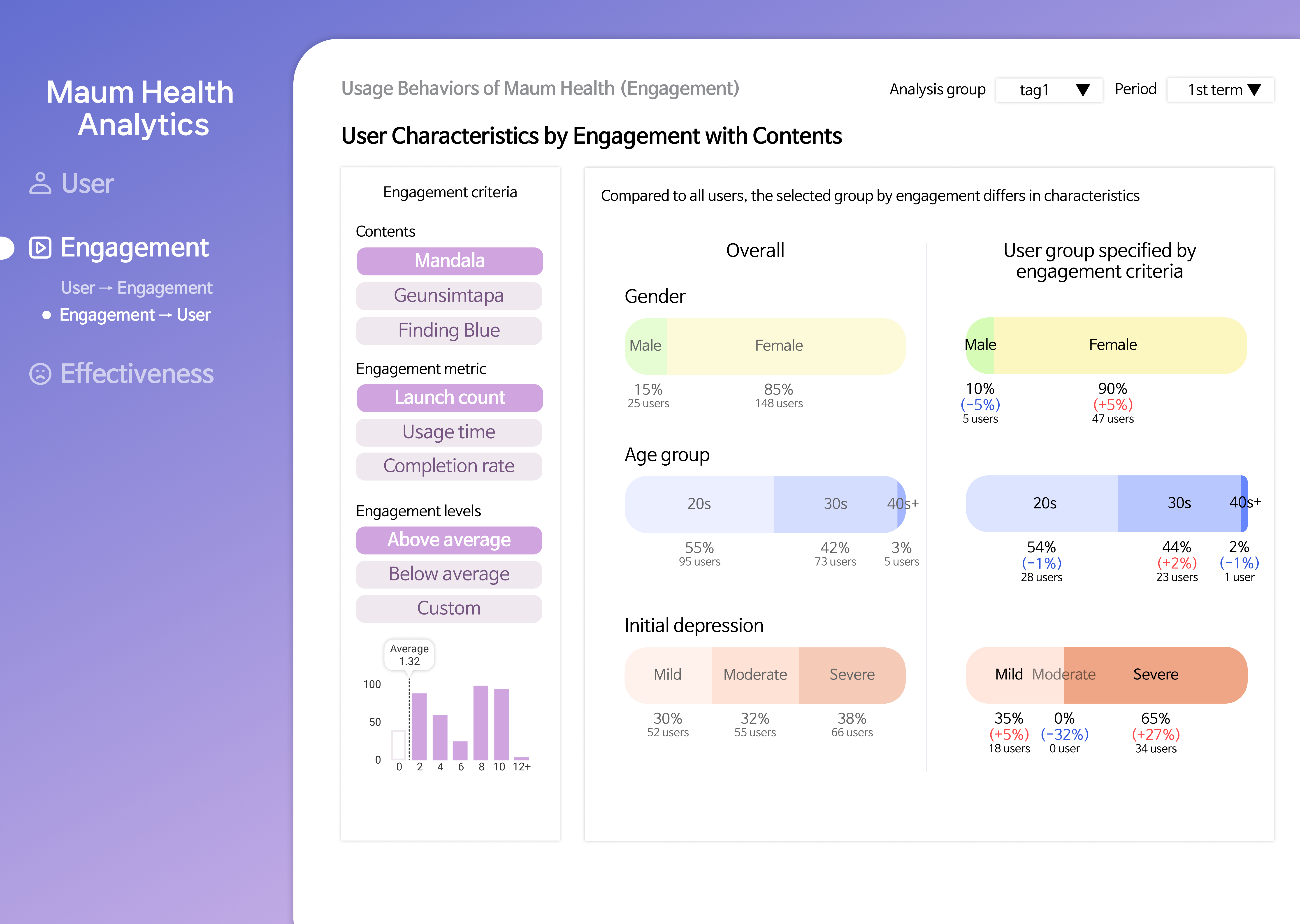


Figure 5. “Engagement” page, exploring the distribution of user characteristics for a user group specified by user engagement metrics

The 'Engagement' page also presents analysis results from a switched perspective. By clicking 'Engagement → User', researchers can investigate how user distribution changes when specifying the user group based on certain engagement levels.

As described in Figure 5, researchers can choose the intervention content to be examined and determine the engagement metric and its range. The range can be selected either based on the mean value (i.e., above or below the mean) or through manual input by the researchers. Then, the distribution of the corresponding users is displayed in the bar chart. As shown on the right side of Figure 5, this distribution is compared with that of the whole user base, indicating how the composition of user characteristics changes depending on certain engagement levels.

## Effectiveness

The 'Effectiveness' page provides the DHI research team with insights into the effectiveness of Maum Health by analyzing changes in depression levels.

### User characteristics, User engagement with Maum Health contents → Effectiveness of Maum Health contents

#### Explore

Similar to the 'Engagement' page, it first allows researchers to explore the effectiveness of a user group through the 'Explore' menu.


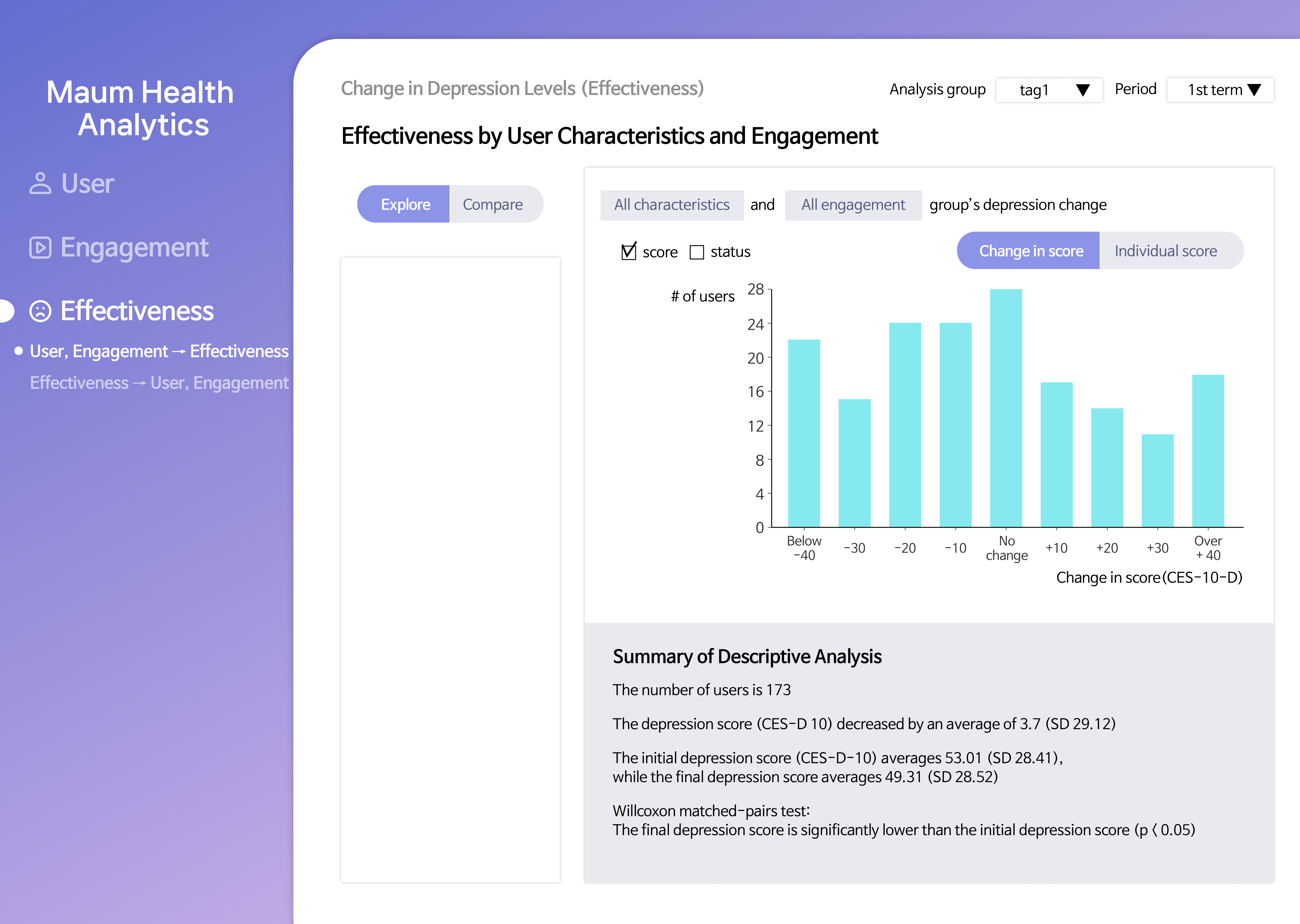


Figure 6. “Effectiveness” page, exploring the distribution of effectiveness for a user group specified by user characteristics and user engagement metrics (using depression scores)

As shown in Figure 6, this page presents the distribution of users based on changes in depression levels using a bar chart. In addition, it can display the distribution of the original scores (i.e., initial and final scores) by changing the view. Below the chart, both descriptive statistics and statistical test results for the change are provided to indicate whether there is a significant change in depressive symptoms.

This distribution can also be illustrated based on depression states (i.e., mild, moderate, and severe) instead of scores, as shown in Figure 7. Using this chart, researchers can quickly overview the overall trends in symptom changes and identify the ratio of users whose symptoms have improved or worsened.

On this page, researchers can specify user groups using both user characteristics and user engagement with Maum Health contents. By default, it shows the effectiveness for all users. However, by selecting a specific group of users (e.g., users with an initial depression state of 'severe' and a Mandala launch count 'above average'), researchers can understand how much the depression levels of this specific group have changed.


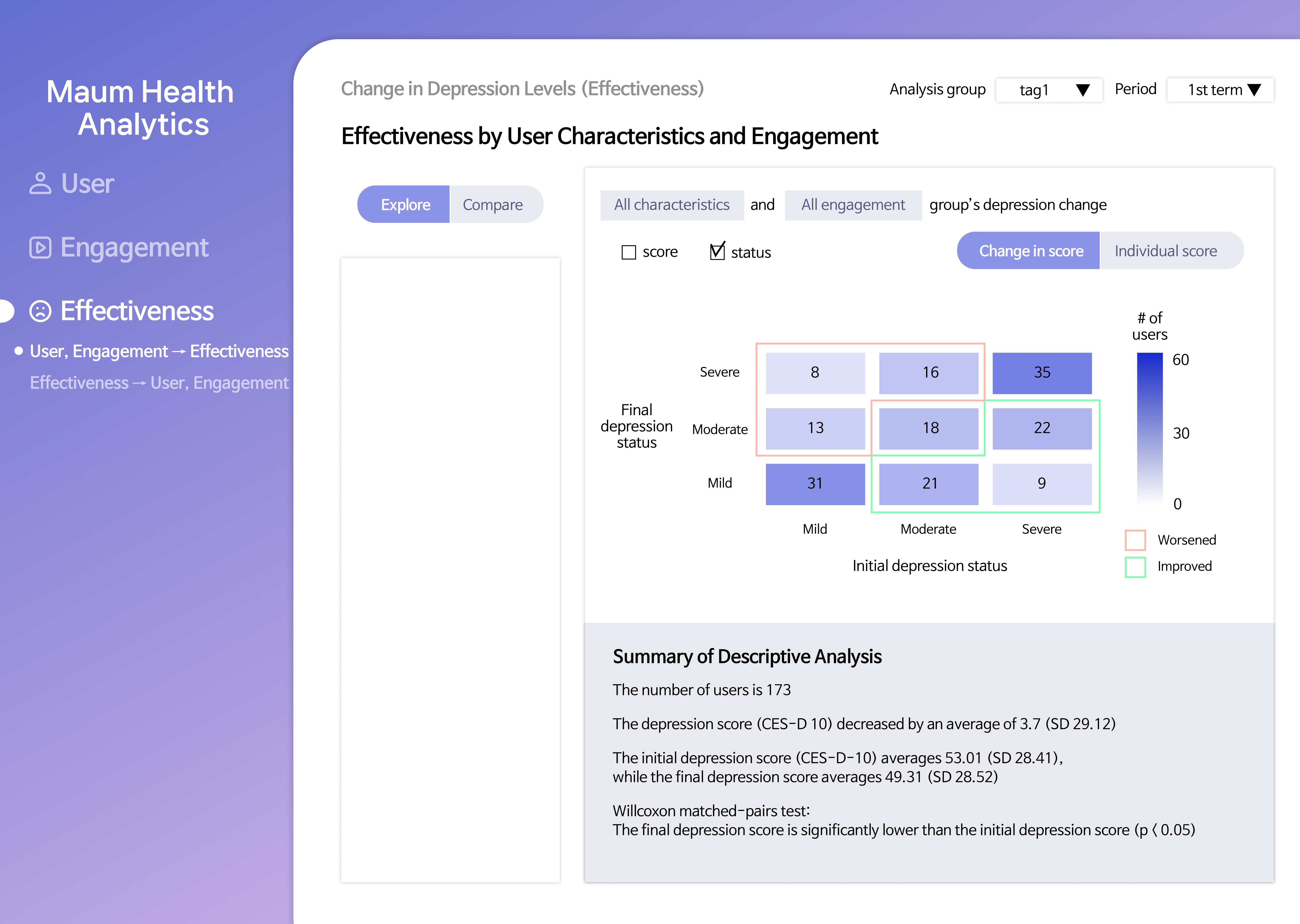


Figure 7. “Effectiveness” page, exploring the distribution of effectiveness for a user group specified by user characteristics and user engagement metrics (using depression status)

#### Compare

The 'Compare' menu of the 'Effectiveness' page allows researchers to compare the effectiveness of DHIs for different user groups. As in the 'Explore' menu, researchers can select user characteristics and engagement level conditions. However, for this menu, the first condition is used as the subgroup criterion, while the latter one is used as the comparison criterion.

For example, as illustrated in Figure 8, researchers can set the analysis target as users whose initial depression state was 'severe' and compare the depression change levels among those users depending on whether the Mandala launch count was above average or not. By following these steps, researchers can investigate how these two conditions affect the effectiveness of DHIs.


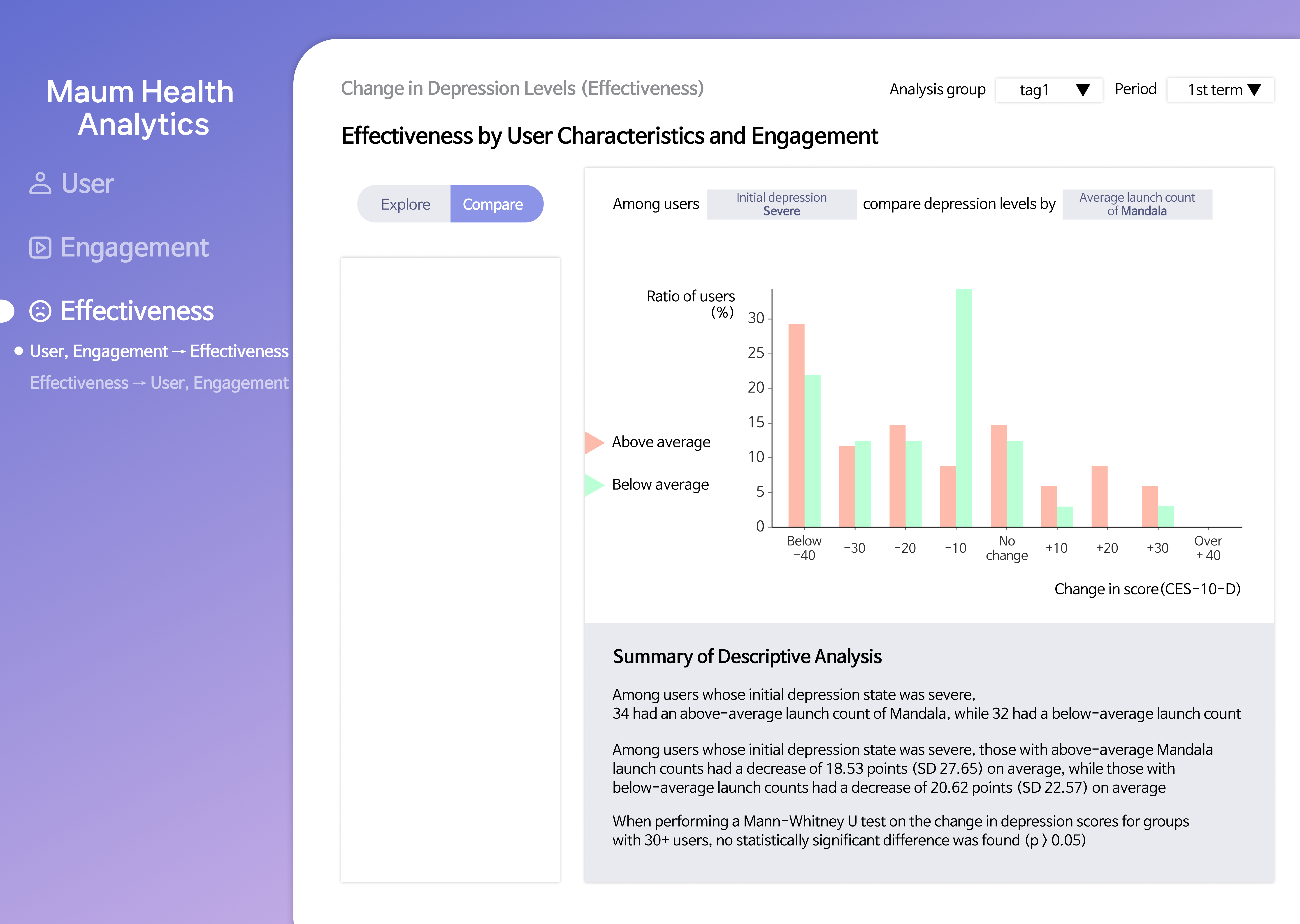


Figure 8. “Effectiveness” page, comparing the distribution of effectiveness between user groups specified by user characteristics and user engagement metrics

### Effectiveness of Maum Health contents → User characteristics, User engagement with Maum Health contents

On the 'Effectiveness' page, researchers can examine how the distribution of user characteristics and engagement levels varies depending on the effectiveness level.

After selecting the 'Effectiveness → User, Engagement' option in the menu, they can choose the level of depression change to form a user group of interest. They can specify the user group based on either changes in the depression state (e.g., decrease, increase, and no change) or by entering a range of depression score changes. In the case of Figure 9, a group of users was chosen whose depression score decreased by 20 to 40 points.

This page then displays the difference in user characteristic distribution and average engagement levels of the chosen user group compared to all users. Based on this information, researchers can identify which user characteristics fall within the target effectiveness range and whether this group shows more active content usage.


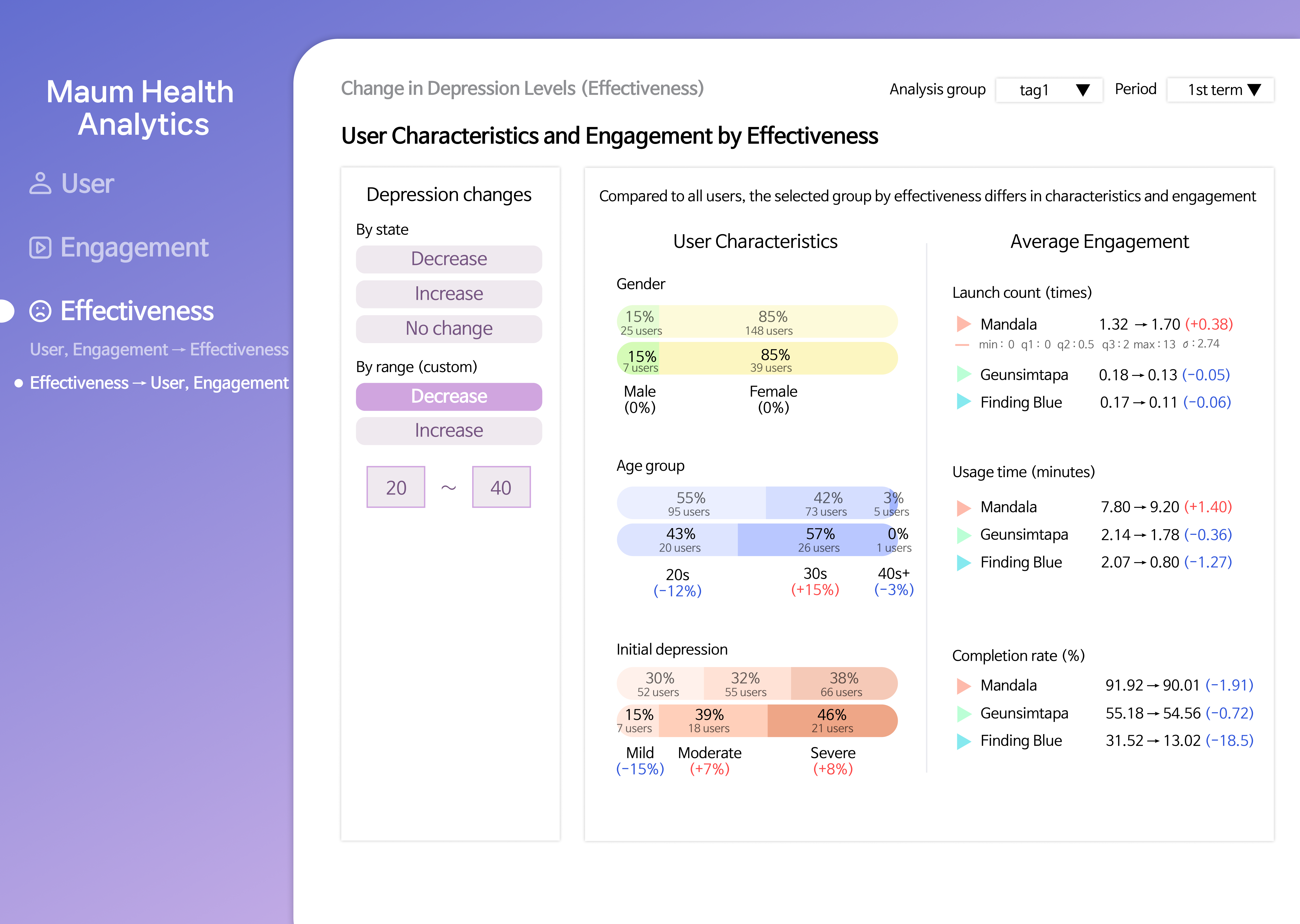


Figure 9. “Effectiveness” page, exploring the distribution of user characteristics and user engagement metrics for a user group specified by effectiveness of DHIs
